# Analysis of The Cancer Genome Atlas sequencing data reveals novel properties of the human papillomavirus 16 genome in head and neck squamous cell carcinoma

**DOI:** 10.18632/oncotarget.15179

**Published:** 2017-02-07

**Authors:** Tara J. Nulton, Amy L. Olex, Mikhail Dozmorov, Iain M. Morgan, Brad Windle

**Affiliations:** ^1^ Department of Oral and Craniofacial Molecular Biology, VCU Philips Institute for Oral Health Research, Virginia Commonwealth University School of Dentistry, Richmond, VA, USA; ^2^ C. Kenneth and Dianne Wright Center for Clinical and Translational Research, Virginia Commonwealth University, Richmond, VA, USA; ^3^ Department of Biostatistics, Virginia Commonwealth University, Richmond, VA, USA; ^4^ Massey Cancer Center, Virginia Commonwealth University, Richmond, VA, USA; ^5^ Department of Internal Medicine, Division of Hematology, Oncology and Palliative Care, Virginia Commonwealth University, Richmond, VA, USA

**Keywords:** HPV16, head and neck cancer, TCGA, integration, genomic structure

## Abstract

Human papillomavirus (HPV) DNA is detected in up to 80% of oropharyngeal carcinomas (OPC) and this HPV positive disease has reached epidemic proportions. To increase our understanding of the disease, we investigated the status of the HPV16 genome in HPV-positive head and neck cancers (HNC). Raw RNA-Seq and Whole Genome Sequence data from The Cancer Genome Atlas HNC samples were analyzed to gain a full understanding of the HPV genome status for these tumors. Several remarkable and novel observations were made following this analysis. Firstly, there are three main HPV genome states in these tumors that are split relatively evenly: An episomal only state, an integrated state, and a state in which the viral genome exists as a hybrid episome with human DNA. Secondly, none of the tumors expressed high levels of E6; E6*I is the dominant variant expressed in all tumors. The most striking conclusion from this study is that around three quarters of HPV16 positive HNC contain episomal versions of the viral genome that are likely replicating in an E1-E2 dependent manner. The clinical and therapeutic implications of these observations are discussed.

## INTRODUCTION

Human papillomaviruses (HPV) are causative in a number of human diseases ranging from simple hand warts, to several types of ano-genital and oral cancers [1]. HPV are non-enveloped double stranded DNA viruses of around 8Kb and their life cycle is intimately linked to the differentiation of the infected epithelium [2]. Following infection the viral DNA enters the host nucleus and transcription from the viral genome is activated by cellular factors resulting in expression of viral genes including the oncogenes E6 and E7 [3]. E7 interacts with Rb to relieve repression of E2F1 resulting in activation of S phase genes and entry into the cell cycle. E6 interacts with p53 resulting in degradation of this protein thereby eliminating the “guardian of the genome”; the result of the oncogene action is an aberrant induction of cellular proliferation [4]. There are three phases of the viral life cycle [5]; firstly, establishment occurs in the basal epithelial cell infected where the viral genome number is controlled at between 20 and 50 copies; secondly, there is a maintenance phase when the infected cell proliferates and begins to differentiate and the viral genome copy number is retained at 20-50 copies; thirdly, there is an amplification stage where the viral genome count increases to up to 1000 copies in terminally differentiated non-proliferating cells, at this stage the viral structural proteins L1 and L2 are produced that then encapsidate the viral genome allowing egress of mature infectious viral particles. HPV has two viral proteins that contribute to replication of the viral genome in association with host cellular factors; E2 is a DNA binding protein that dimerizes and recognizes 12bp palindromic sequences surrounding the viral origin of replication and recruits the viral helicase E1 to the origin via a protein-protein interaction [6, 7]. E1 interacts with components of the host DNA polymerase machinery in order to initiate replication of the viral genome [8, 9].

In HPV16 positive cervical cancer there is a proportion of cases in which the viral genome has integrated into that of the host with the loss of the viral E2 gene and such cancers have increased DNA copy number imbalances [10]. Combined with the observations that loss of E2 promotes cellular growth of HPV containing cells in vitro [11, 12], it has been proposed that loss of the E2 gene during viral integration promotes cervical cancer development via increased expression of the E6 and E7 oncogenes. While there have been many investigations into the viral genome status in cervical cancer the physical status of the viral genome in HPV positive oral cancers has been less well studied. In recent years, there has been a large increase in the incidence of HPV positive oropharyngeal carcinomas, the number of which are increasing annually [13], though, HPV positivity is an indicator of better overall survival in head and neck cancer [14]. HPV positive head and neck cancer cell lines have an increased sensitivity to radiation, which may explain the enhanced survival of HPV positive patients [15]. With the availability of The Cancer Genome Atlas, there is now an opportunity to investigate in greater depth the status and expression of the HPV genomes in HPV positive head and neck cancer. Previous work investigating 279 head and neck cancers identified 35 that had high risk HPV16, 31 and 33 and indicated that 25 of these tumors had the viral genome integrated into that of the host [16]. One of the observations made in this report was that for some of the integrated tumors the viral genome integrated and then excised as a viral-human hybrid sequence that then replicated autonomously. The availability of TCGA data and the intriguing observation that the viral genome replicates autonomously as a hybrid with human DNA prompted us to investigate the structure of the viral genome, and RNA expression from the genome, in HPV positive head and neck cancers in more detail.

Analysis of the data determined that of the 520 HNC samples analyzed, 72 were HPV positive as determined by HPV RNA analysis. To our knowledge, this is the first report that has analyzed all cases of head and neck cancer sequencing data from TCGA. The large majority of the HPV positive tumors had HPV16 present so this virus type was the focus of our study. The data reported here presents several novel results. 1) Our evidence suggests that almost three quarters of tumors retain episomal viral DNA, some of which replicate along with human DNA as viral-human hybrid episomes. 2) The presence of partially deleted HPV genomes plus full length genomes in tumors previously has been interpreted as indicating both integrated viral genomes plus episomal viral genomes existing in the same tumor, however, our analysis indicates that this interpretation may not be correct for HNC. Our results show that such a pattern demonstrates that the virus is maintained as a dimer/multimer or as a hybrid with human DNA with one part of the viral genome missing in one or more of the viral genomes present in the HPV16 dimer/multimer. 3) There is very little expression of full length E6 transcript, the dominant E6 present is E6*. E6*I does not have the ability to target p53 for degradation therefore it is possible that these tumors retain a responsive p53 protein that could contribute to improved response of HPV positive versus negative head and neck tumors [14]. 4) There is no correlation between viral genome load and RNA expression. We propose that conclusions from previously published studies describing the status of the HPV genome in human cancers should be re-evaluated given the results presented here.

## RESULTS AND DISCUSSION

### HPV types present in head and neck cancer

The data used within our analyses were generated by the TCGA Research Network: http://cancergenome.nih.gov/ We analyzed 520 HNC samples (referred to as HNSC by TCGA) for the presence of HPV based on the raw data for TCGA RNA-Seq (Supplementary Table 1 lists the HPV genomes used for screening and Supplementary Table 2 details the HPV types present in the TCGA samples). 72 of 520 samples were positive for HPV, summarized in Table 1. HPV16 is detected in 83% of HPV-positive HNC with HPV33, 35 and 56 also being detected. These percentages are in alignment with other reports [13, 17].

**Table 1:** HPV Presence in HNC Samples

Overall HPV Incidence	
14% (72/520)	
Type	% of HPV-positive
HPV16	83.3
HPV33	11.1
HPV35	4.2
HPV56	1.4

### HPV16 genome structural status and gene expression in HNC

**Figure 1:**
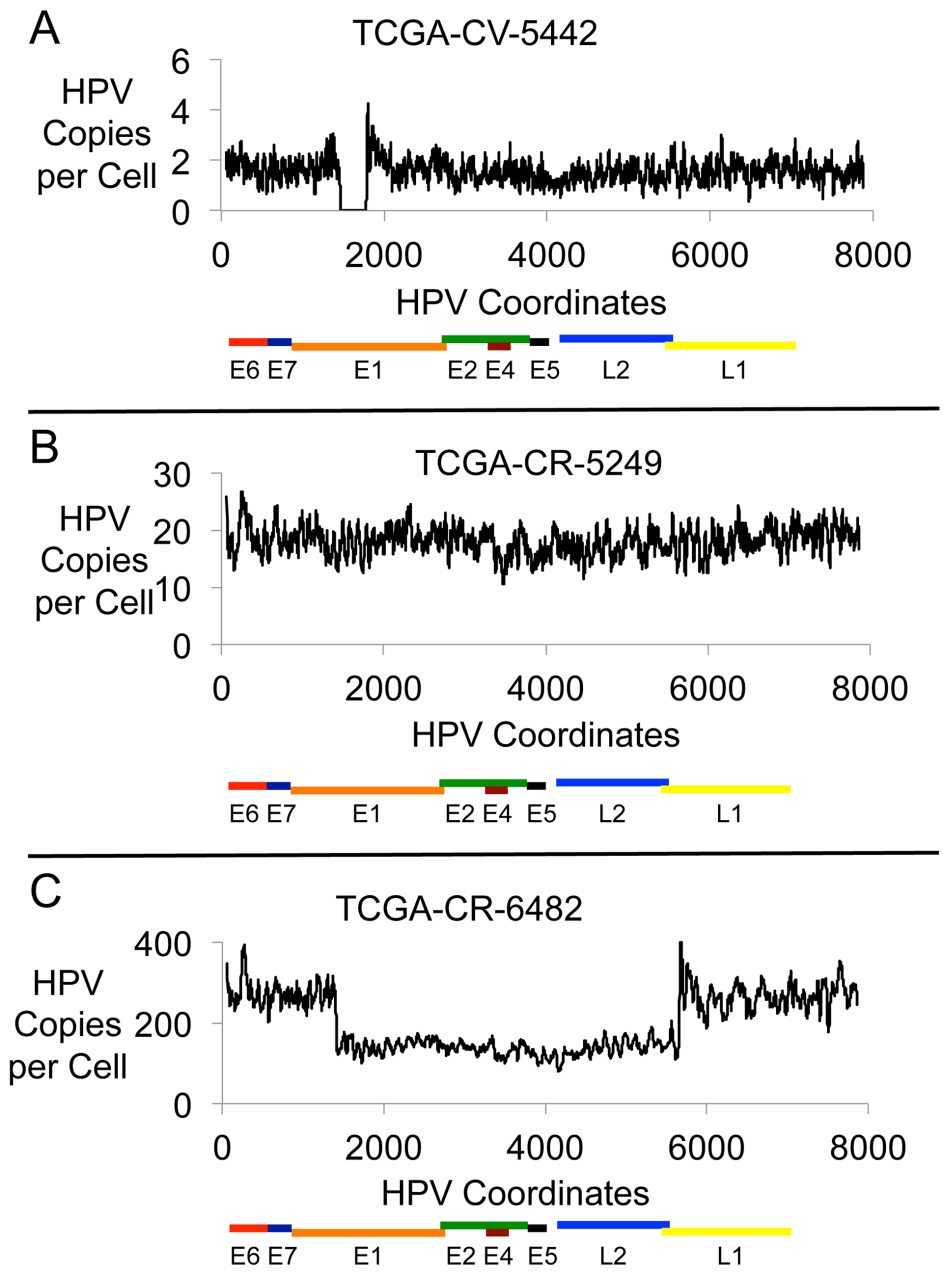
HPV16 Genomic DNA Profiles Plots are shown for three HNC samples with HPV16 DNA showing the HPV DNA present in each sample in copies per cell. The results displayed are based on the content for a 20-base interval across the 7904 bp HPV16 genome. The three samples are: **A**. TCGA-CV-5442, **B**. TCGA-CR-5249, and **C**. TCGA-CR-6482.

We compared the HPV genomic structure and HPV gene expression for HPV16 positive HNC samples. The paired end DNA sequence reads that aligned to the HPV16 genome were quantitated for 30 HNC samples (all of the available samples for HPV16 positive HNC from TCGA Whole Genome Sequence (WGS) data). The mapping coverage for these samples ranged from 17X to 141X with an average of 64X coverage. Profiles of viral copy number across the HPV genome were generated for the samples and three typical patterns were identified; representative profiles for each pattern type are shown in Figure 1, representing three categories based on HPV genomic and gene expression profiles. The categorization of samples was based on HPV DNA structure as well as RNA expression patterns (described later). Figure 1A shows an HPV genome with a partial deletion (Category 1). Figure 1B shows a profile with a fairly constant copy number across the entire HPV genome (Category 2). Figure 1C shows an HPV genome that has a partial deletion yet not in all copies of the HPV genome; intact HPV genomes are also present, generating a profile with a distinctive “half step down, half step up” pattern (Category 3). The representation of these categories in the 30 HNC samples is 7 (23%) for Category 1, 13 (44%) for Category 2 and 10 (33%) for Category 3. Supplementary Figure 1, Supplementary Figure 2 and Supplementary Figure 3 show the HPV16 viral genome DNA copy number and RNA profiles for the 30 tumors for Category 1, 2 and 3 respectively. Supplementary Table 3 lists the tumor samples assigned to each category according to our analysis.

### Category 1 tumors have a reduced viral load compared with Categories 2 and 3

Reaching a high viral genome copy number could readily occur for episomal HPV because the HPV genome can be replicated via E1 and E2 in a more than once per cell cycle manner [2, 18]. However, if the HPV genome is integrated into the human genome with complete loss of E1/E2 expression then there would be no ability to use the HPV origin, and an increase in copy number might not readily occur. We examined the HPV copy number for the three categories of the HPV structural status in HNC samples and the results are shown in Table 2. The average copy number for Category 1 is 3.5 and a median of 1.7, while the average copy number for Category 2 samples is 20.5 with a median of 14.7 (the difference between Category 1 and 2 being statistically different with a p-value of 0.0008). Category 3 had a mean copy number of 57.5 with a median of 18.5, which is also a statistically significant difference from Category 1 samples (p-value of 0.0012). There is no statistically significant difference between the viral genome copy numbers in Category 2 and 3 tumors. This analysis suggests that Category 1 samples have integrated HPV while both Category 2 and 3 samples have the HPV genome in episomal form.

**Table 2:** HPV Copy Number in Three Categories of HNC Samples

	HPV genome copy number for each Category			*p*-value for overall comparison	*p*-value for each comparison		
Category	1	2	3		1 *vs* 2	1 *vs* 3	2 *vs* 3
Mean	3.5	20.5	57.5	0.0019	0.0008	0.0012	0.34
Median	1.7	14.7	18.5				

### Category 1 tumors have HPV16 genome structural and expression patterns consistent with integration

**Figure 2:**
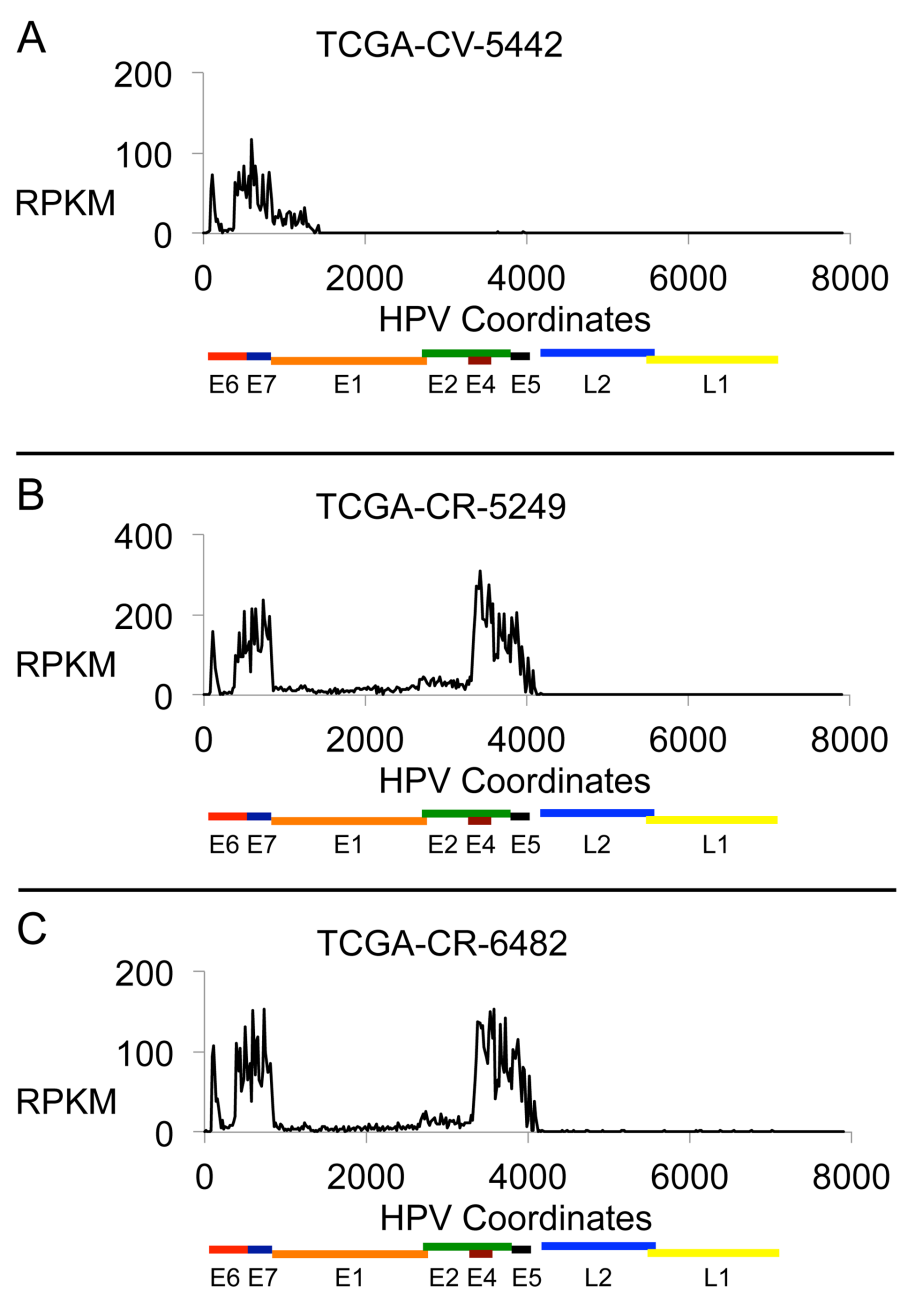
HPV16 RNA Expression Profiles Plots are shown for three HNC samples with HPV16 DNA showing the HPV RNA sequence expression in each sample in units of Reads Per Kilobase per Million (RPKM). The results displayed are based on the content for a 20-base interval across the 7904 bp genome. The three samples are: **A**. TCGA-CV-5442, **B**. TCGA-CR-5249, and **C**. TCGA-CR-6482.

Analysis of profiles from RNA-Seq data (Figure 2) was performed. Category 1 samples are defined not only by a genomic deletion pattern, but by a characteristic gene expression pattern in which the E6 and E7 gene transcripts are produced but expression through the E1 or E2 gene is truncated such that there is no or little expression of E1, E2 or in most cases downstream genes E4 and E5. For Category 1 samples, the truncated RNA expression pattern represented the over-riding pattern for categorization. This is shown in Figure 2A, which shows the RNA-Seq profile for the DNA sample shown in Figure 1A. The sites of HPV deletion correspond to the sites of HPV recombination with the human genome as determined by the presence of viral-human hybrid DNA fragments (Supplementary Table 4). This is the expected expression pattern for an HPV genome that has integrated, deleted DNA in the E1 to E2 region, and dislocated upstream promoters, such as the HPV LCR promoter, from genes downstream of the HPV site of integration, thus eliminating transcription past the integration site.

### Category 2 tumors have HPV16 genome structural and expression patterns consistent with viral episomes

The gene expression profile from RNA-Seq that helped define Category 2 samples shows transcript sequences that run through E1, E2, and past E4 to the end of E5 (Figure 2B; this is the RNA-seq data from the sample shown in Figure 1B). The level of transcripts for E1 and E2 are relatively low compared to those for genes E4 and E5. Analysis of RNA splice junctions through this region revealed that this relatively low level of E1 and E2 was primarily due to RNA splicing at positions 880^3358 to express the E1^E4 protein [19]. There were no consistent and significant levels of viral-human hybrid fragments present in these tumors (a significant level was determined by having 0.5 copies per cell or higher), though there were some sporadic junction fragments indicating that there may be some viral integration in a few cells in the tumor. Therefore, we propose the predominant form of the HPV16 genome driving these tumors from Category 2 is episomal.

### Category 3 tumors have HPV16 genome structural and expression patterns consistent with the presence of partially deleted viral and viral-human hybrid episomes

Category 3 tumors have a “half step down, half step up” pattern in viral genome copy numbers (Figure 1C). The viral RNA expression that helped define Category 3 tumors is very similar to that for Category 2 tumors and an example is given in Figure 2C (the RNA expression data from the tumor used in Figure 1C). This shows that the expression levels of the RNA encoding viral replication factors (E1 and E2) are no different between Category 2 and Category 3 tumors demonstrating these tumors have the capacity to initiate replication from the HPV origin.

An explanation for the pattern of viral DNA in these samples could be that the viral genome exists as a dimer, trimer, tetramer etc. and has lost a portion of one or more of the viral genomes. There is evidence for the existence of such multimeric HPV genomes in tumors harboring episomal viral genomes [20–22]. An alternative is that these tumors contain a mixture of episomal and integrated viral genomes as found for cervical cancer samples [22]. Many of the Category 3 tumors contain an approximately equal number of intact versus deleted genomes, in a 1:1 ratio, regardless of the copy number (Table 3). This 1:1 association is statistically significant when compared to what one would expect by chance (p-value = 0.0008). It is unlikely for an intact HPV genome and a deleted HPV genome to co-exist while maintaining similar copy number in so many samples and not be physically connected. This supports the idea that many tumors have the deleted and intact genomes covalently linked as dimers or multimers.

**Table 3:** Copy Number of Intact and Deleted HPV Genomes in HNC Samples

HNC Samples	BA-4077	CR-6482	CV-5971	CR-7385	CV-5443	CN-5374	BA-A4IH	CR-6470	CR-7404	HD-7754
**intact:deleted copies**	11:14	138:130	4:5	15:8	9:75	11:20	6:6	6:7	41:42	118:104
**HPV-Human DNA Junction Copy Number**	12	143	5	ND	31	4	0	0	0	0

ND indicates the HPV-Human DNA junctions were not easily quantifiable due to the complexity of the junctions.

To further investigate these tumors, the ratio of intact to deleted genome numbers per cell along with the number of hybrid HPV-human fragments per cell were determined and the results shown in Table 3. There were four tumors (BA-A4IH, CR-6470, CR-7404, HD-7754) in which there were no specific HPV-human junction fragments and an approximate 1:1 ratio of intact to deleted genomes. We propose that these tumors contain viral episomal genomes of dimers or multimers in which there is a partial deletion in half of the genomes present on the episome. For another three tumors (BA-4077, CR-6482, CV-5971) there existed an approximate 1:1 ratio of intact to deleted viral genomes but also an approximate 1:1 ratio of HPV-human junction fragments to intact HPV genomes. The data suggests that in these tumors the viral genome exists as an episome of viral (a genome dimer or multiple thereof with half of the genomes containing a deletion) and human DNA. If an HPV multimer were integrated into the human genome, we would expect only one copy of the HPV-human junction fragments. Tumor CN-5374 had an approximate 1:2 ratio of intact to deleted viral genomes and an HPV-human hybrid fragments to intact viral genome ratio of 1:3. E1-E2 is still expressed in this sample, therefore, we favor a model where there are multimers of the HPV genome in which two thirds of the viral genomes have lost a section but that the viral genomes replicate as a hybrid viral-human episome. In CR-7835, there is a 2:1 intact to deleted viral genome ratio indicating the possible presence of a trimer, or multiple thereof, which contained a deletion in one copy of the viral genome per trimer. In this case, it was impossible to determine a clear number for the HPV-human hybrid fragments due to the complexity of the junction. CV-5443 had an approximate ratio of intact to deleted genomes of 1:8 and an intact HPV-human hybrid count of approximately 1:3. It is possible that this tumor has a complicated viral genome structure consisting of multimers of viral genomes containing a deletion in around one eighth of the genomes replicating as episomes with human sequences. It is also possible that there is a genuine mixture of integrated and episomal viral genomes in this sample.

It is important to note that in the past, ratios of E2 to E6 DNA of less than 1 has been used to indicate that the viral genome is integrated into that of the host in cervical cancer. We suggest that this may not an accurate indicator in HNC, and that in fact the presence of E2 DNA at a ratio to E6 of ~0.5 in a tumor more consistently predicts the presence of a HPV-human hybrid episome or the presence of a multimer of HPV genomes replicating in an E1-E2 dependent manner. To investigate this further we studied in depth BA-4077, CR-6482 and CV-5971; these were the tumors that had an approximate ratio of 1:1 for intact to deleted genomes and a ratio of approximately 1:1 for intact HPV-human hybrid fragment counts (see Table 3). Evidence has also been shown supporting an episomal HPV-human DNA structure existing in BA-4077 [16], although this report did not address the incidence of these types of structures in HNC.

### Evidence supporting the presence of viral-human hybrid episomes in HNC tumors

**Figure 3:**
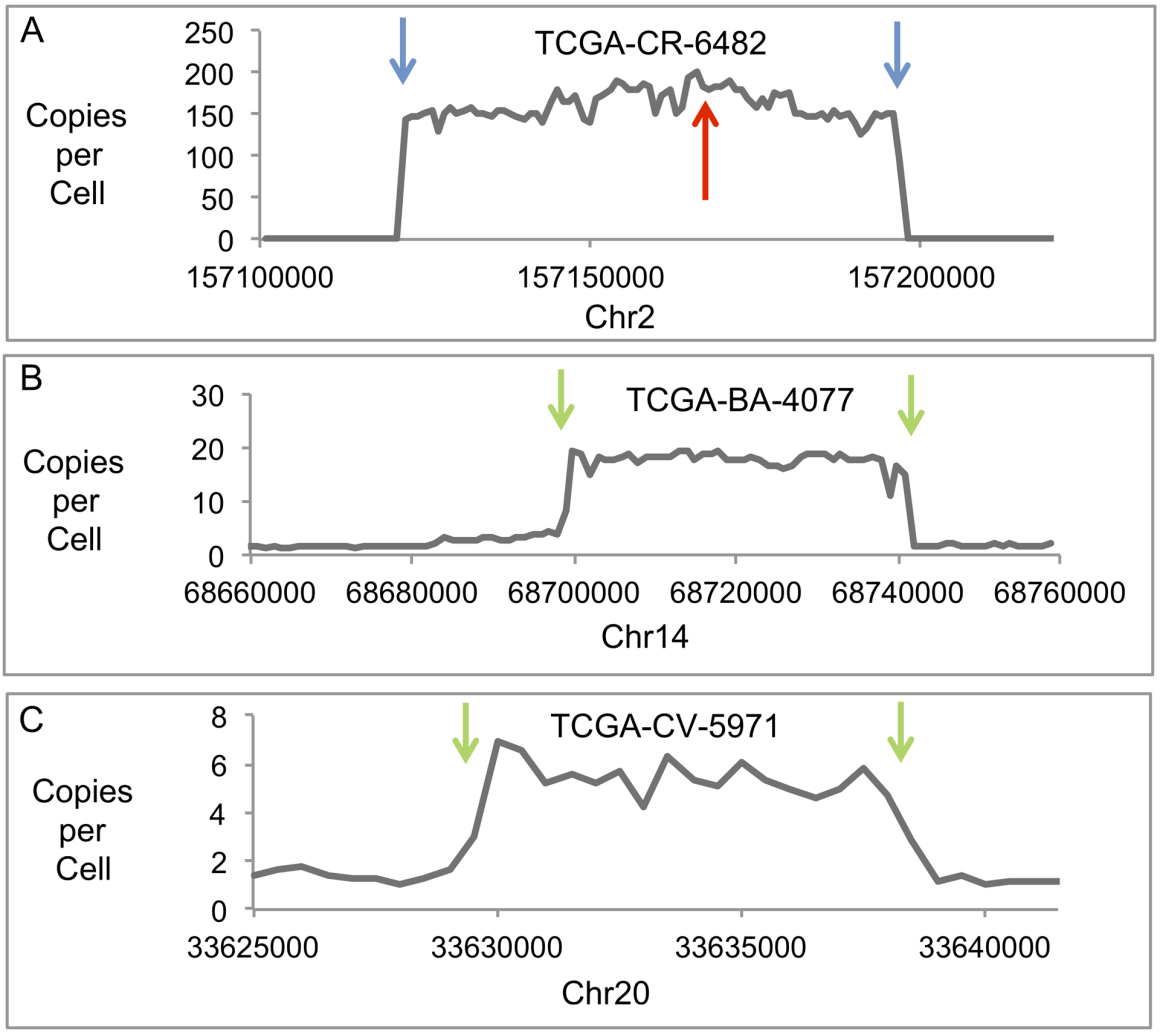
Human Genomic DNA Profiles of Amplified Regions Plots are shown of copies per cell for three human DNA regions with junctions to HPV DNA and amplification in three different samples. The results displayed are based on the DNA content for a 1000-base interval across the genomic region shown for TCGA-CR-6482 and TCGA-BA-4077, and a 500-base interval for TCGA-CV-5971. **A**. Plot shows a region of Chr2 amplified in TCGA-CR-6482. The red arrow indicates the site of HPV recombination with human DNA and junctions between HPV and human DNA. The blue arrows indicate the sites for the ends of the human DNA amplicon and the sites that have recombined with each other to form an excision junction. **B**. Plot shows a region of Chr14 amplified in TCGA-BA-4077. The green arrows indicate the sites of the ends of the human DNA amplicon and the sites that have recombined with HPV DNA to form HPV-human DNA junctions. **C**. Plot shows a region of Chr20 amplified in TCGA-CV-5971. The green arrows indicate the sites for the ends of the human DNA amplicon and the sites that have recombined with HPV DNA to form HPV-human DNA junctions.

Sample CR-6482 has 138 intact copies per cell of HPV16 and 130 copies of HPV16 with a partial deletion (see Figure 1C). For the deleted HPV genome, there are two HPV-human DNA junctions at Chr2:157166456-HPV16:1434 and Chr2:157166441-HPV16:5626 with a deletion of 4188 bases from HPV, and 14 bases deleted from the human sequence (human coordinates are based on HG19; Supplementary Tables 6-8). The mapping of these junctions was based on the structure of 12965 human-HPV hybrid fragments, and 6021 human-HPV hybrid reads. The deletion of DNA in the HPV genome can be seen in Figure 1C. The region of human DNA amplified along with HPV DNA was from Chr2, from approximate positions 157121900 to 157197600, a 75 Kb region. Figure 3A shows a profile of this amplified region, which is comparable to that already published [16]. This region, amplified to 160 copies, encodes the entire gene NR4A2, a steroid-thyroid hormone-retinoid receptor family member. Consistent with this amplification, the CR-6482 sample has the highest RNA expression of NR4A2 of the 520 HNC samples (66 fold higher expression than the mean of all samples, Supplementary Table 5). The HPV-human DNA junctions are consistent with the integration of the HPV genome into the human genome. However, we have proposed that the HPV-human DNA from Category 3 samples is within an episome, therefore, we analyzed whether the HPV genome joined to human DNA could have been excised from the human genome to form a circle containing HPV and human DNA. Mapping throughout this region identified the HPV-human genome junctions already described plus a human-to-human DNA junction consistent with an excision and ligation forming a circular structure that could then amplify as an episome. The human DNA excision junction on the proposed circular structure was identified as DNA from Chr2 at positions 157197643/157122001 (Supplementary Table 9 and Supplementary Table 10), which are coordinates that correspond to the two ends of the human DNA amplicon, and present at a copy number of 143, comparable to the copy number for the human amplicon.

**Figure 4:**
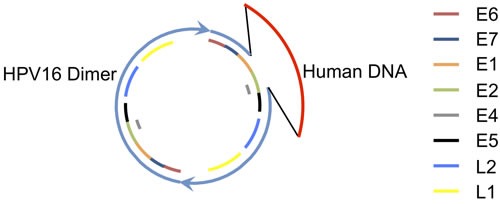
A Proposed Structure for the HPV Dimer Human DNA Hybrid Episome A circular structure with HPV16 and human DNA is shown. Two copies of HPV DNA are linked as a dimer in direct tandem, shown in blue. A partial deletion in one copy of the HPV genome is shown as a gap. Human DNA, shown in red, is joined to the HPV DNA at the sites of the partial deletion. Regions for the HPV genes are shown as color-coded bars.

No integration junctions between the HPV-human hybrid DNA structure and the human genome at any location were found at the one-copy or higher level, therefore, we conclude that the HPV-human hybrid DNA structures are episomal. The odds of missing an integration junction due to gaps in read alignment, such as from bias in sequencing or low coverage, was very low. The largest alignment gap within a 100,000 bp of unamplified sequence near this region was 20 bases, with an average read alignment gap size of 1.4 bases. There was no gap for sequence coverage. The read length was 101 bases, far above a 20 base alignment gap, thus, a junction could not easily be missed by mapping the reads or fragments. We calculate the odds of missing a junction in mapping reads are 0.006 based on the coverage that was below 0.5 copies per cell (the lower limit set for integration detection). These results do not discount the possibility that there is a low level of integration for a subset of structures. The human and HPV DNA mapped as a circle with the bulk of the human DNA region and HPV DNA not joined to any other human DNA locations, therefore, we conclude that a circular structure was formed with the HPV and human DNA. Figure 4 shows the general structure proposed for the hybrid HPV16 and human DNA episome in which a dimer of HPV DNA is shown with the two copies of the HPV genome in direct tandem. A partial deletion is shown in one HPV genome spanning the E2 gene, while the other copy is intact. Human DNA is shown as an insert attached to the HPV DNA at the sites of the partial deletion. We next investigated if there was a deletion within the human genome that corresponds to the human DNA that was excised. There were no aberrant human-human DNA fragments detected with coordinates coinciding with the excision sites that would have formed following excision.

**Figure 5:**
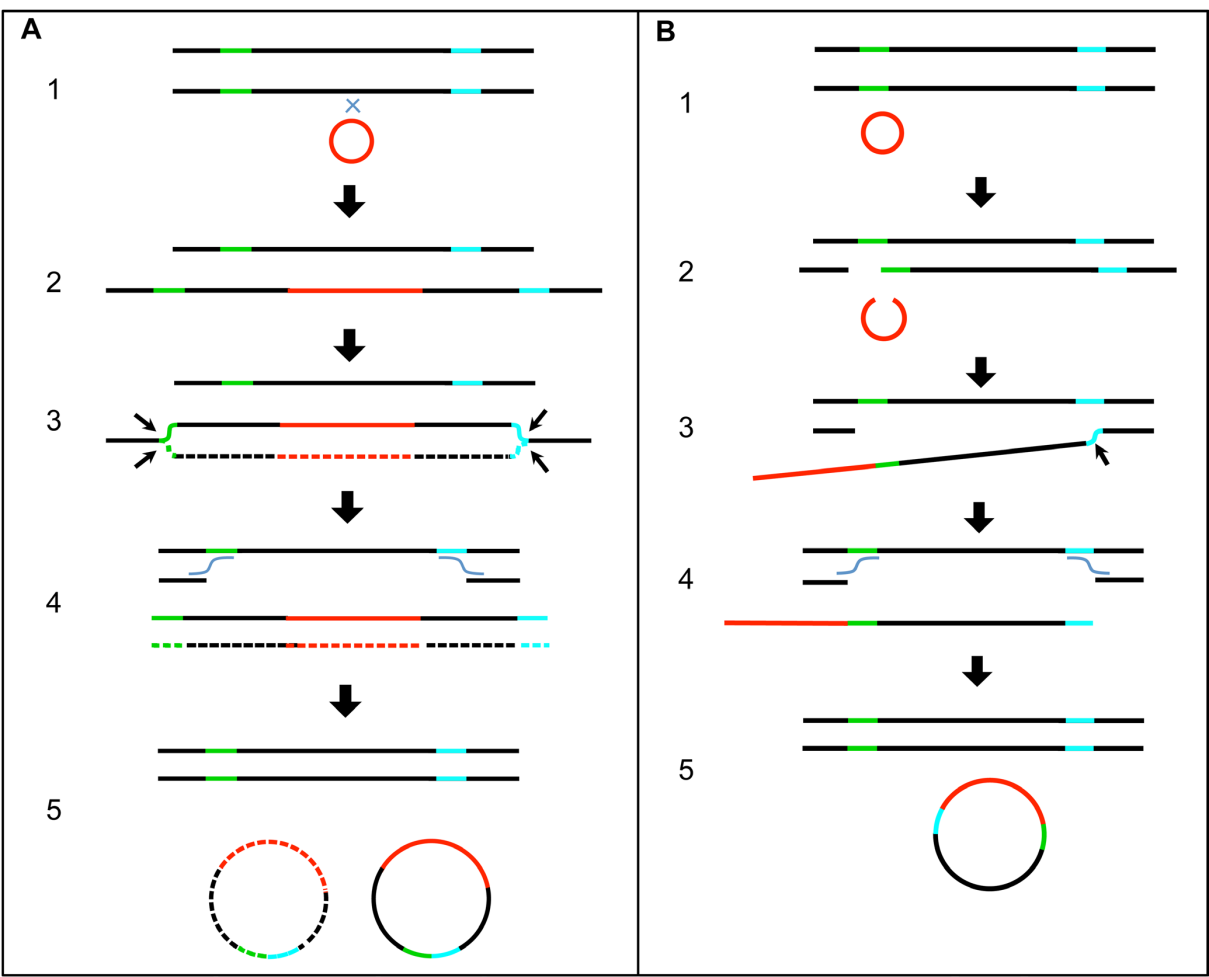
Proposed Mechanistic Models of HPV Recombining with the Human Genome and Excising **A**. A diagram with a series of steps showing the integration of the HPV genome into the human genome, unscheduled replication from the HPV origin to form a replication bubble, excision of the HPV genome along with human DNA to form a circle, and homologous recombination repair of the human genome. The green and blue colored regions on the human DNA represent sites of excision and human-to-human DNA end joining. **B**. A diagram with a series of steps showing breakage of human and HPV genomic DNA, joining of HPV and human DNA, excision of HPV and human DNA to form a circle, and homologous recombination repair of the human genome. The green and blue colored regions on the human DNA represent sites of breakage and joining of human DNA to HPV DNA. Single lines represent double-stranded DNA. Black lines represent human genomic DNA. Red lines represent HPV genomic DNA. Small black arrows mark the sites of DNA breakage and excision. Wavy lines indicate DNA crossovers.

We propose a mechanism for the formation of this structure in Figure 5A. The circular HPV genome first integrates into the human genome by illegitimate recombination. The site of integration in the human genome is within DNA that has already replicated and thus there are two copies of human DNA that form the sister chromatids. HPV integrates into only one of those copies of human DNA (steps 1-2). The second step is replication initiation within the HPV genome from the HPV origin that is dependent on the presence of E1 and E2 expressed from an intact copy of HPV DNA. This creates an aberrant replication bubble structure with replication branches that will not progress far since the human DNA has already finished replication (step 3). The cell resolves this aberrant structure by cleaving at the replication forks releasing the HPV DNA flanked on both sides by human DNA (step 4). This also creates a damaged broken chromosome that could conceivably be repaired by homologous recombination using the intact sister copy of human DNA, thus leaving no evidence of a deletion. The ends of the freed HPV and human DNA are repaired by non-homologous end joining (NHEJ) to form a circle (step 5). There are numerous variations possible in the steps in the proposed mechanism. This straightforward model when applied to an HPV dimer accounts for the production of a circular dimer episome with an intact HPV genome capable of expressing E1 and E2, and a partially deleted HPV genome attached to human DNA. The expression of E1 and E2 promotes unscheduled replication initiation from the viral origin of replication within the HPV-human hybrid episome, which leads to amplification of HPV and attached human DNA.

BA-4077 has two major junctions mapped at chr14:68699686-HPV16:917, and chr14:68741694-HPV16:3435 (Supplementary Table 11–Supplementary Table 13), with secondary junctions present at a low incidence. These junctions were detected and mapped based on 755 junction fragments and 230 junction reads. The human DNA joined to the HPV DNA is a 42-Kb region (see Figure 3B) amplified to a level comparable to the copy number for the intact and partially deleted HPV genomes and the human-HPV DNA junctions (Table 3). This is comparable to results published by Parfenov [16]. These two major junctions cannot be the result of integration, excision, and ligation as proposed for CR-6482 in Figure 5A because there were no aberrant human-human hybrid fragments detected that would explain the joining of two distal human sequences as detected for CR-6482 (the green-blue excision junction in Figure 5A). Instead, two distal human sequences that flank both ends of the amplified human DNA are joined to HPV DNA. The mapping of the HPV and attached human DNA is consistent with a circle. There was no deletion detected within the human genome that coincided with this 42 Kb excision. No integration junctions between the HPV-human hybrid DNA structure and the human genome at any location were found at the one-copy or higher level, therefore, we conclude that the HPV-human hybrid DNA structures are episomal. The largest gap in read alignment within a nearby unamplified 100,000 bp region was 117 bases with an average read alignment gap size of 4.8. Thus, a junction could be missed by mapping the 101 base reads. We calculate the odds of missing a junction in mapping reads are 0.014. In addition, the 42Kb fragment encodes an exon for the Rad51B gene and the BA-4077 expresses the highest level of Rad51B transcripts of all 520 samples (Supplementary Table 5). Figure 5B describes a mechanism that explains the viral-host genome hybrid detected in BA-4077. The first step is a break in a viral episomal structure followed by re-section of some of the viral DNA resulting in deletion. One end of this broken viral genome is then ligated to host DNA using NHEJ (steps 2-3). Following ligation the viral genome then undergoes replication in an E1-E2 dependent manner that promotes stress on the host DNA and a second break in the host genome (steps 3-4). The resulting viral-host linear DNA is ligated by NHEJ to form a viral-host hybrid episome that can replicate in an E1-E2 dependent manner, and the host genome is repaired using homologous recombination (step 5). It should be noted that in the BA-4077 sample in the viral-human episome there is an internal deletion within the E1 gene that results in complete loss of E1 expression (see Supplementary Figure 1), therefore the replication origin for this episome may be within the human sequence co-replicating with the viral genome. This deletion could have happened following the formation of the viral-human episome. Alternatively, the deletion could have occurred during the recombination with the human sequence in which case there would be an E1-E2 independent mechanism for generating the stress and breakage in the viral genome. It is also possible that alternative sequences in the HPV viral genome are acting as origins of replication [23–25].

A third sample (CV-5971) was analyzed and found to have an ~8 Kb region of human DNA from chr20 joined to HPV DNA and together amplified to ~5 copies (see Figure 3C). This region encodes two exons of the TRPC4AP gene, though its RNA expression level was not unusually high in this sample compared to all samples. The structure of this DNA was similar to that of BA-4077, in that the two opposite ends of the human DNA that eventually is amplified were joined to two ends of HPV DNA. The two junctions are chr20:33629921-HPV16:881 and chr20:33638720-HPV16:6456 (Supplementary Table 14-S15). These junctions were detected and mapped based on 130 junction fragments and 17 junction reads. There were no integration junctions detected between any HPV-human DNA structures and the human genome at the one-copy or higher level, therefore, we conclude that the HPV-human hybrid DNA structures are episomal. The largest gap in read alignment for a nearby unamplified 100,00 bp region was 121 bases with an average alignment gap size of 6.0. Thus, a junction could be missed by mapping the 51 base reads, but less so by fragment mapping. We calculate the odds of missing a junction in mapping reads are 0.078. Mapping of this DNA is consistent with HPV16 plus human DNA having formed a circle. Again, there was no evidence of an associated deletion of the 8 Kb human DNA region, and one possibility is that it was repaired using homologous recombination, though, other mechanisms are feasible. We propose the mechanism of formation for this DNA structure is similar to that described in Figure 5B for BA-4077.

Our conclusion from the analysis of these three samples is that despite the apparent HPV genome integration into the human genome based on HPV-human DNA recombination junctions, the HPV DNA and joined human DNA is episomal and the HPV genome was only transiently joined to the human genome before being excised to form a circular extrachromosomal structure. Of the three structures analyzed, the first sample's structure appeared to be derived from an integration and subsequent excision, but we propose that for the second and third samples’ structures, the HPV DNA was never formally integrated but merely recombined with human DNA through DNA breakage and repair events. These results demonstrate that HPV DNA attached to human DNA in at least some of the Category 3 samples exists episomally as a viral-human hybrid and not as an integrated structure.

### High expression of the alternatively spliced E6*I transcript compared to the intact E6 transcript

Analysis of the RNA-Seq data for HPV gene expression data and RNA splice junctions for HNC samples revealed that in addition to the splice junction that results in the expression of the E1^E4 protein, E6 is spliced to form the protein variant E6*l (226^409) [26]. This is evident from the gap in the E6 profiles in Figure 2, and found for all HPV-positive samples regardless of Category. The expression level of intact E6 was an average of 9-fold lower than the level of E6*l, with E6*l levels being comparable to E7 levels. This is similar to the dominance of E6*1 expression in cervical cell lines [27, 28]. E6*I is not known to exhibit p53 interactions and mediated degradation of p53 as known for intact E6 [29]. Therefore, the role of E6*I in HNC may manifest through known E6*I functions, such as induction of oxidative stress in the cell [30]. It is possible that during the initial infection full length E6 is indeed expressed to combat p53 to allow establishment of the viral infection. One proposed reason that HPV+ HNC respond better to radiotherapy than HPV- tumors is an induced p53 protein level that exceeds the capacity of E6 in response to treatment [15].

### No correlation between E7 or E6 expression levels and HPV copy number

**Figure 6:**
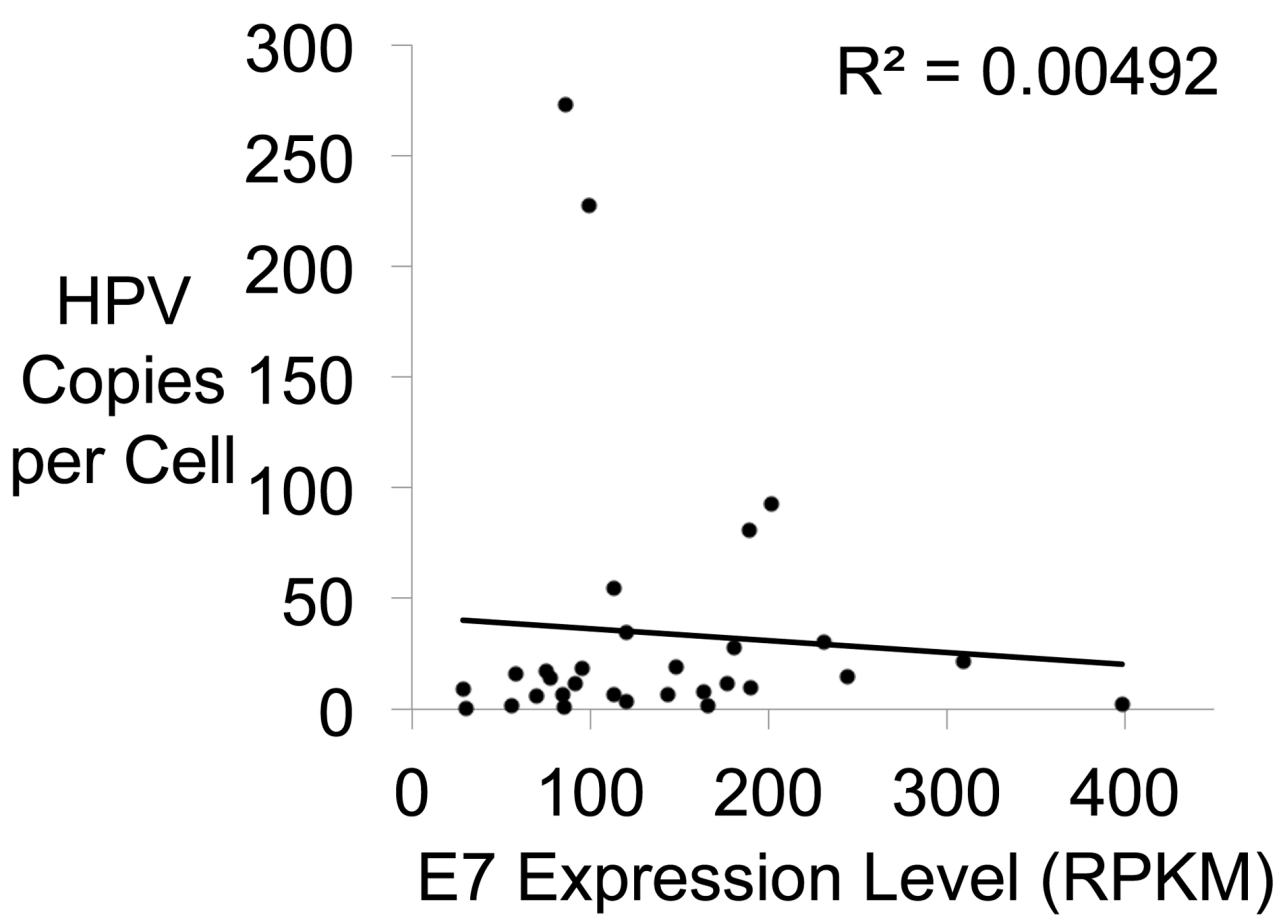
E7 Gene Expression Versus HPV Copy Number A plot showing no relationship between HPV copy number and E7 gene expression. Copy number per cell and E7 gene expression level in each HNC sample were plotted. The R^2^ shows no significant correlation.

HPV genomes are maintained at multiple copies per cell, which potentially could impact the expression levels of E6 and E7. The whole genome analysis shown in Figure 1 provided assessment of the copy number of HPV genomes per cell. We plotted the copy number of the E6/E7 region of the genome versus the E7 expression level and found no correlation between the number of copies of the E7 gene and the level of E7 transcripts produced (Figure 6). We conclude that HPV copy number does not affect the expression levels of E6 and E7, and therefore these genes are regulated independent of copy number. These results corroborate other studies of HNC and cervical cancer that showed a lack of correlation between HPV gene expression and viral load [31, 32].

## SUMMARY

In this report, we show there are three main head and neck tumor types in relation to HPV16 genome status; integrated only, viral episomes only, and viral-human hybrid episomes. There may also be others where there is a mixture of integrated and episomal viral DNA although this would be a minor category. For the first time, this report demonstrates the presence of hybrid viral-human episomal genomes in a large proportion of HPV positive head and neck cancers. There is a large body of work investigating the status of HPV16, particularly in cervical cancer, and the conclusions from these studies have been that there are three main categories of tumors in relation to HPV16 genome status; tumors with integrated, episomal, and mixed (integrated plus episomal) [33–38]. Assuming our results are representative of the larger population, we propose that this last group has been mischaracterized in HNC and that theses tumors are predominantly viral-human or partially deleted dimer episome-containing tumors. The evidence to support this conclusion is as follows. First, the fragments containing the viral-human reads are similar in number to the number of fragment reads for the viral genome present in the tumor. If there were integrated plus episomal HPV then it would be expected that there would be much fewer viral-human fragments compared with the viral genome. Second, in the three tumors analyzed in detail and described here, the human DNA that is predicted to be amplified if a viral-human episome existed is indeed amplified to a level similar to the viral genome. Third, the presence of viral-human hybrid episomes has already been demonstrated for the sample BA 4077. This was done using a FISH probe for the proposed human DNA present in the viral-human episome in which punctate staining was observed in the original tumor tissue nuclei [16]. Fourth, Category 2 and 3 tumors have statistically similar viral genome copy numbers present while Category 1 (truly integrated) have statistically fewer than either 2 or 3 supporting the conclusion that Category 3 tumors indeed have episomal genomes present. It is important to note here that the demonstration of the viral-human hybrid episomes is compatible with the work done by others in cervical cancer where these tumors were characterized as mixed episomal and integrated HPV16 tumors [33–38]. In fact, most of these studies support our findings. An E2 to E6 ratio of less than 1 was often used to confirm the mixed status (although the actual ratio is often not given, it is just stated that it is less than 1); our Category 3 tumors in general have such a ratio.

In many studies, an E2 to E6 DNA ratio of greater than zero and less than 1 was used to determine the mixed status of episomal and integrated HPV [33–38]. Our results for HNC samples suggest that this approach would indicate in many cases, the presence of episomal HPV with deletions. What seems problematic with the existence of cells with both integrated and episomal HPV genomes is that the expression of E1 and E2 from the episome could initiate unscheduled replication from the integrated HPV origin [18]. However, it is possible that such mixed tumors exist as has been proposed for cervical cancer. We propose that the simple lack of detection for E2 RNA (or even E4 or E5 RNA, which has a better signal to noise ratio) could be an improvement over and alternative to DNA analysis for characterizing the tumor as being an integrated tumor. Such a simple characterization could be used to definitively determine whether there is a worse outcome in patients with HPV+ HNC that have integrated HPV genomes versus those with HNC with episomal HPV, which would include episomal HPV-human hybrid DNA. A recent analysis of HPV positive tonsillar and base of tongue cancers showed expression of E2 in 64% of tumors aligning with our data [39].

We propose that 73% of tumors (Categories 2 and 3) from HNC have HPV episomes replicating in an E1-E2-dependent manner, therefore, direct targeting of E1-E2 could be of therapeutic advantage as it would reduce the viral load resulting in reduction of E6 and E7 expression. Loss of E6 and E7 is known to induce apoptosis in HPV positive cancer cell lines, and would affect a much larger fraction of tumors than previously thought [40–43]. Inhibitors of the E1-E2 interaction exist for low risk HPV types [44, 45], therefore, in principal, such an inhibitor could be developed for disrupting HPV16 E1 and E2 interaction to promote cell death of the infected cancer cell.

Future work should focus on enhancing our understanding of the contributions of amplified human DNA to the head and neck cancer phenotype and better characterizing those tumors that have truly integrated viral DNA to determine whether they have a worse outcome as they do in cervical cancer. It is also possible that such viral-human hybrid episomes exist in some cervical cancers and this should be investigated. Perhaps the most striking outcome of the data presented here is the potential to target E1-E2 DNA replication for therapeutic gain in three quarters of HPV16 positive head and neck cancers. Such targeting should be prioritized.

## MATERIALS AND METHODS

### Analysis of sequencing data

WGS and RNA-Seq BAM files with read alignment to HG19 for HNSC samples were downloaded from NCI's Cancer Genomics Hub [46]. Most samples were sequenced with 101 base reads while a small subset of HPV16 positive samples (2) were sequenced with 51 base reads. An in-house pipeline was developed to extract all unmapped reads from BAM files and re-align them to 171 HPV genomes (see Supplementary Table 1 for list). This pipeline includes the following steps, which are described in detail below: prepare HPV reference genome file, download and perform quality control on TCGA BAM files, extract unmapped read pairs, convert unmapped read pairs to FASTQ format, align unmapped read pairs to the HPV reference genomes (see Supplementary Methods for more details).

HPV Reference Genome Construction: The FASTA-formatted genomes of 171 HPV types were downloaded from PaVE (http://pave.niaid.nih.gov/; [47]) on March 9, 2015. These FASTA files were concatenated into one file and indexed using the bowtie2-build command [48].

Extracting Unmapped Reads: All BAM files obtained from CGHub for both RNA-Seq and WGS data consisted of paired-end data. Samtools [49] was used to extract the unmapped reads. The merged BAM file containing unmapped reads was then converted to FASTQ format. This step was done differently for RNA-seq data and WGS files due to formatting issues with the RNA-seq data. WGS files were converted to a paired set of paired-end FASTQ files using the PICARD v1.115 tool SamToFastq.jar (http://broadinstitute.github.io/picard). RNA-seq files were converted to single-end FASTQ files using the BEDTools2 bamToFastq script [50].

Re-alignment to HPV genomes: WGS and RNA-seq FASTQ files were aligned to the composite HPV reference genome file using BowTie2 [48] with the additional options “end-to-end” and “very-sensitive”. In addition, RNA-seq FASTQ files were treated as unpaired data by using the “-U” option.

### General genome mapping and visualization

Select mapping of regions of HPV and human DNA was accomplished using UGene [51]. Profiles of RNA and DNA reads for HPV were based on summed read counts for a window size of 20 bases, with a 1-base sliding window across the genome coordinates.

### Copy number determination for human and HPV DNA

The HPV copy number calculation for genomic plots was based on the following. We determined the read count for HPV16 DNA per 20 base interval throughout the HPV genome. The expected average read count for a 20 base interval if there was one copy per cell was calculated as total human read counts per sample for itself and mate mapped / (number of bases for a human haploid genome / interval size). The copy number is thus the ratio of the read count for HPV16 per interval to the expected read count for a single copy. Therefore, copy number = read count for HPV16 DNA per 20 base interval / (total reads per sample/(3.23×10^9^/20)). A total copy number for HPV was based on the average over thousands of bases in regions of interest. The same approach was used for copy number determination for amplified human DNA.

DNA junction copy number based on junction fragments was determined in a similar manner by junction fragment count / (total fragments per sample/ (3.23×10^9^ / average HPV16 fragment size)). Results of junction copy number based on reads was more variable than that based on DNA fragments because reads cover a shorter distance than the fragments that cover a few hundred bases. The shorter the region, the more likely the results are to be affected by sequencing bias. This variability can be seen within the HPV genomic plots. Also, there were substantially more data for junction fragments than for junction reads, therefore, our analysis of junction copy number was based on fragments. We note that copy numbers for junctions based on fragment analysis is still more variable than copy number based on many thousands of bases used in the HPV and amplified human DNA quantitation. In addition, alignment for human DNA was performed by TCGA while the HPV alignment was performed independently within this study. Therefore, quantitation of copy number for human DNA may not be entirely congruent with quantitation of copy number for HPV DNA.

Limits of detection for HPV-human DNA junctions based on hybrid fragments was dependent on fragment mapping coverage. The junction sites need not be located within the region of each fragment that is sequenced. Mapping coverage was based on the total number of fragments and fragment size, which is generally greater than sequence coverage.

### Comparing HPV genome copy number between Category 1, 2, and 3 samples

Categories 1, 2 and 3 were compared for sample HPV genome copy number using the non-parametric Kruskal-Wallis H Test, and in a pairwise analysis using the non-parametric Mann-Whitney U Test.

### Gene expression analysis for HPV16

The HPV16 gene annotations with gene coordinates was obtained from RefSeq [52]. Reads with mapped coordinates corresponding to regions specific for each gene were counted. The probe region specific to E4 overlaps a portion of E2, and thus E4 expression quantitation also includes some signal from E2. However, the levels of E2 were low in all cases, and thus we regarded the E2 contribution as having minimal effect on the E4 levels. E2 expression was quantitated in a region non-overlapping with E4. We report the quantitation as the reads per kilobase of defined transcript region per total million reads (RPKM) for each gene.

HPV RNA splice junctions were identified by using Tophat 2 [53], as well as analysis of unmapped reads in which 5’ and 3’ end 15 base subsequences aligned to the HPV16 genome and mapped to coordinates with distances between ends greater than the read length.

### Human gene expression analysis

RNA-SeqV2 data was downloaded from the TCGA data portal (https://tcga-data.nci.nih.gov/tcga/), and data from rsem.genes.normalized_results files were used unmodified for gene expression quantitation.

### DNA junction analysis

Human-HPV DNA junction fragments were identified by sets of paired-end reads in which one read mapped to the human genome and the other end read mapped to the HPV genome. The lists of junction fragments were comprehensive and used for quantitation. The minimum number fragments used in identifying HPV-human junctions was 11. In addition, quantification of these junction fragments had to be greater than the 0.5 copies per cell limit to be regarded as a potential single site of integration. Reads that cover the junction were identified by defining a median position of junction fragments using the human DNA coordinates (Location 1). The identification was accomplished by first extracting read pairs in which one read mapped to either human or HPV DNA and the other paired read was unmapped to either HPV or human DNA. A 4000 base human DNA sequence (Sequence 1) centered on Location 1 was used for mapping a subsequence of the unmapped reads. A 15-base subsequence for the 5’ end and the 3’ end from the unmapped reads was aligned to either Sequence 1 or HPV DNA. Reads that had 5’ and 3’ ends aligned to human and HPV were identified as junction reads. Additional reads overlapping the precise human and HPV DNA junctions were identified in some cases to provide additional mapping data particularly for junctions that were complex, e.g., when a single read did not include both HPV and human DNA due to the presence of extraneous DNA. The precise coordinates of the junction for the human and HPV genome were determined from these reads.

Human DNA excision and deletion junctions were identified first by extracting paired end reads that mapped to coordinates within 2.5 Mb of the human coordinates for human-HPV junctions and had a distance between paired reads of greater than 900 bases (the size of the largest sequencing fragment). The reads that then clustered to within 900 bases of each other were selected for identification and mapping of read junctions as already described and fragments quantified. Deletion junctions were distinguished from excision junctions by having the correct sequence order within the aligned sequence (lower coordinates before higher coordinates). The excision junctions were identified as having the reverse sequence order (higher coordinates before lower coordinates).

The Human-HPV DNA junctions with HPV coordinates that were consistently within 450 bases of an internal partial deletion within the HPV genome were considered linked and the deletion proposed to be associated with the HPV-human DNA recombination.

### Comparing distribution of deleted and intact HPV genomes to that expected by chance

We modeled the copy number distribution expected based on the observed copy number, ranging from 4 to 138 for intact HPV and 5 to 130 for deleted HPV by a randomized permutation simulation. We then calculated the relative mean absolute differences for values in this model, and compared this to the actual relative mean absolute differences for the samples based on the observed. The non-parametric Mann-Whitney U-test was used to assess the statistical significance between the two populations.

## SUPPLEMENTARY MATERIALS FIGURES AND TABLES




