# Supplementary material for: Analysis of The Cancer Genome Atlas sequencing data reveals novel properties of the human papillomavirus 16 genome in head and neck squamous cell carcinoma
Source: Oncotarget. 2017 Feb 7;8(11):17684–99. doi: 10.18632/oncotarget.15179 (PMC5392278; doi:10.18632/oncotarget.15179)
Supplement: Supplementary file 1 [file oncotarget-08-17684-s001.pdf]

Analysis of The Cancer Genome Atlas sequencing data reveals novel properties of the human papillomavirus 16 genome in head and neck squamous cell carcinoma

Supplementary Material

TCGA cohorts
Sequencing and clinical data was downloaded from the Cancer Genomics Hub (CGHub) between March 2 and June 17, 2015. For RNA-seq and WGS data the GeneTorrent-3.0.113 software was used to identify and download the requested BAM files for HNSC cohorts. Additional details for each TCGA cohort follow.

HNSC RNA-seq: On March 2, 2015 GeneTorrent-3.0.113 software tools were used to identify and download TCGA RNA-seq BAM files for HNSC from CGHub. The cgquery tool identified 524 HNSC samples using the following query string to obtain all primary tumor HNSC RNA-seq BAM files aligned to the HG19 human genome assembly: "study=phs000178&library_strategy=RNA-Seq&disease_abbr=HNSC&sample_type=01&refassem_short_name=HG19&state=live". Download was attempted on April 13, 2015. Four files had been suppressed by CGHub, therefore, these samples were removed from all further analyses, which left 520 HNSC RNA-seq primary tumor samples.

HNSC WGS: On April 16, 2015 GeneTorrent-3.0.113 software tools were used to identify and download TCGA whole genome sequencing (WGS) BAM files for HNSC from CGHub. The cgquery tool identified 173 HNSC WGS samples using the following query string to obtain all primary tumor HNSC WGS BAM files: "study=phs000178&sample_type=01&state=live&disease_abbr=HNSC&library_strategy=WGS&filetype=bam". Of the 173 samples, there were only 158 unique patient IDs while 15 patients had two associated files. These samples were marked and remained in the analysis for verification purposes in down-stream analyses.

BAM file processing: RNA-seq and WGS BAM files were downloaded from the Cancer Genomics Hub (CGHub). The SAMTools 'flagstat' program (samtools version 0.1.18) was run on each BAM file using the command line "samtools flagstat $infile >> $outfile", to gather various statistics of number of total reads, mapped reads, properly paired reads and unmapped reads.

Extracting unmapped reads: All BAM files obtained from CGHub for both RNA-seq and WGS data consisted of paired-end read data. The "samtools view" program was used to extract the unmapped read pairs for each sample into a temporary file using the following command-line options, respectively: "-f 4 –F 264", -f 8 –F 260", "-f 12 –F 256". Each temporary file was sorted by name with the command "samtools sort –n", and then all files for each sample were merged into one BAM file using the command "samtools merge –n".

The merged BAM file containing unmapped reads was then converted to FASTQ format. This step was done differently for RNA-seq data and WGS files due to formatting issues with the RNA-seq data as described on CGHub's website [https://cghub.ucsc.edu/ts/dnashortread.html. WGS files were converted to a set of paired-end FASTQ files using the PICARD v1.115 tool SamToFastq.jar. RNA-seq files were converted to single-end FASTQ files using the BEDTools2 bamToFastq script.

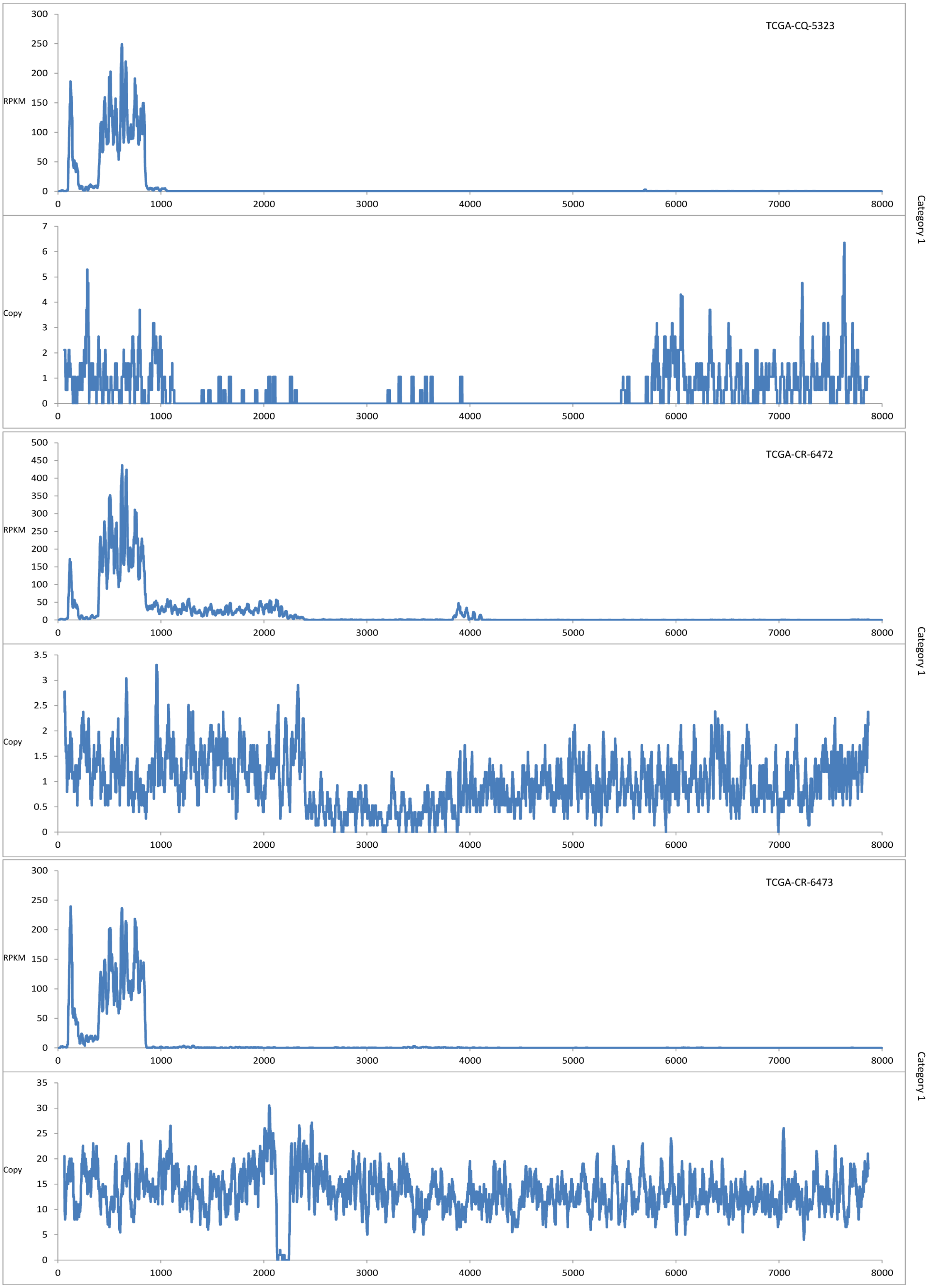
TCGA-CQ-5323
RPKM
Copy
Category 1
TCGA-CR-6472
RPKM
Copy
Category 1
TCGA-CR-6473
RPKM
Copy
Category 1
0
1000
2000
3000
4000
5000
6000
7000
8000

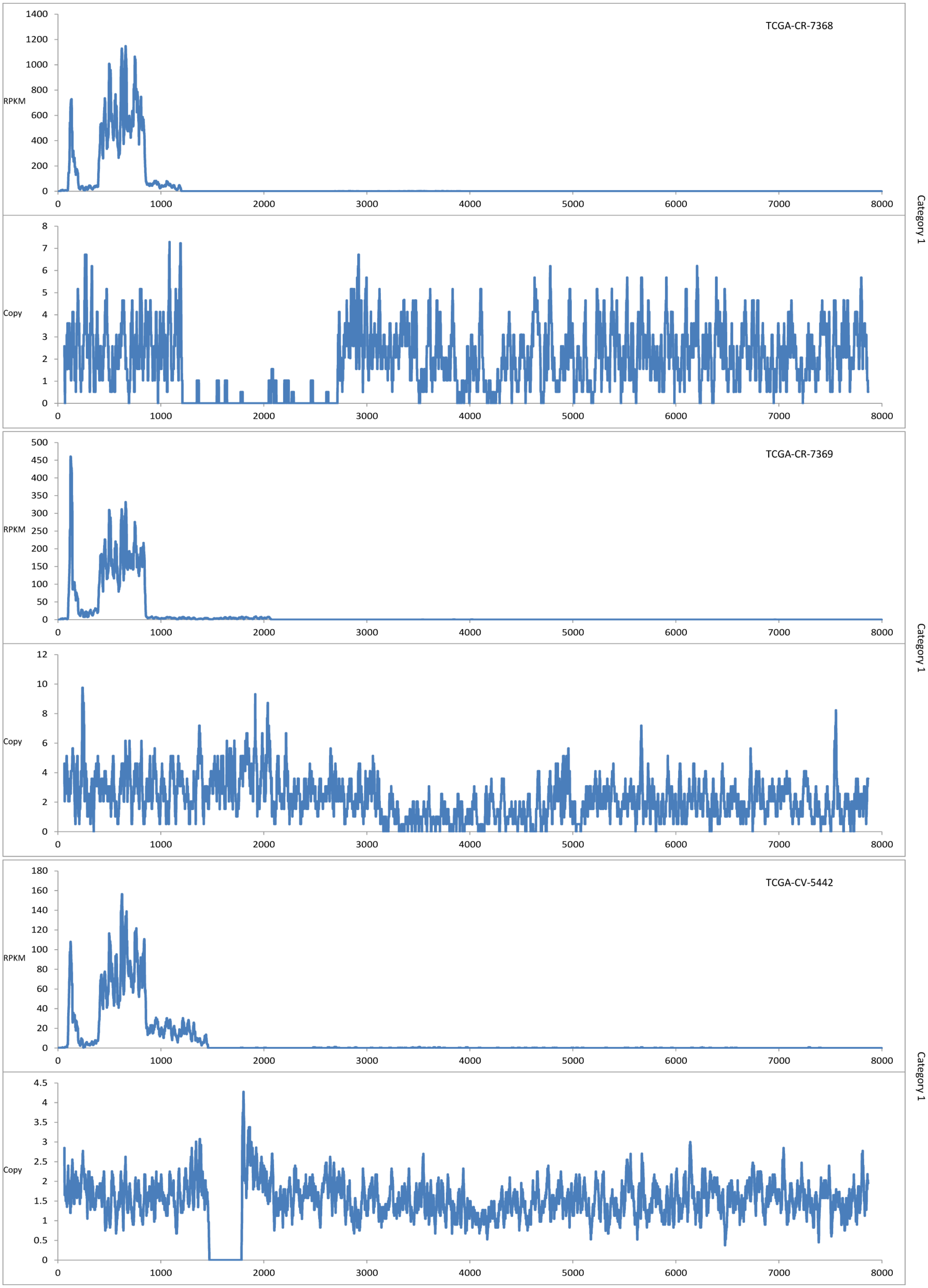
TCGA-CR-7368
RPKM
Copy
TCGA-CR-7369
RPKM
Copy
TCGA-CV-5442
RPKM
Copy
Category 1
Category 1
Category 1

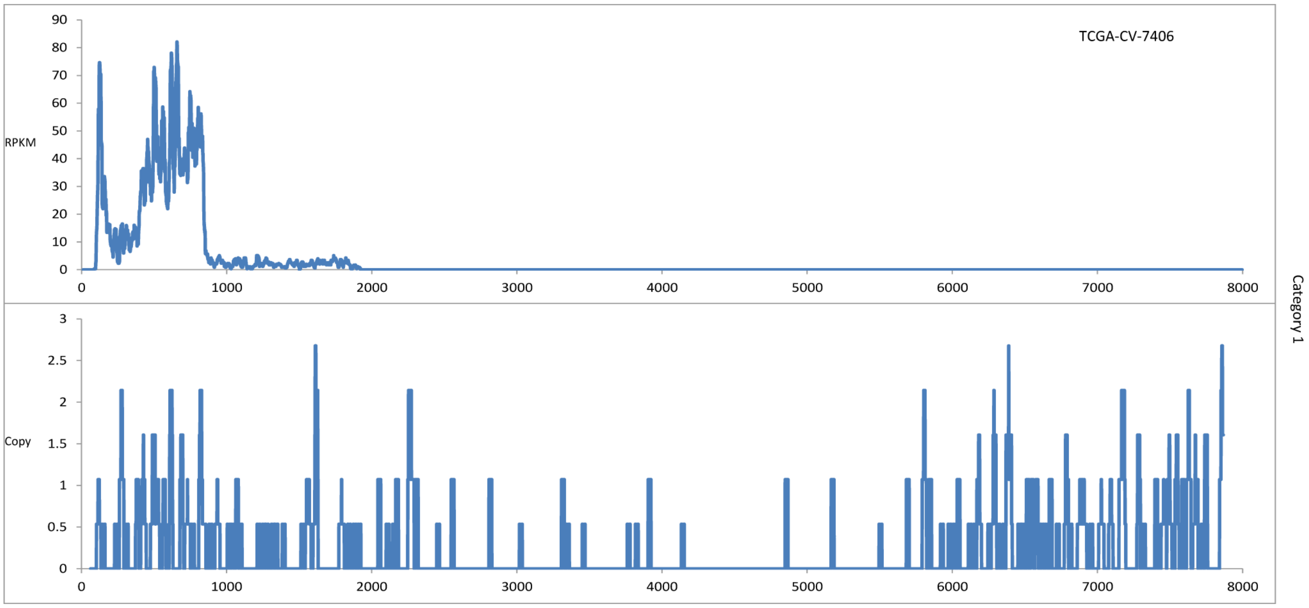

Figure S1: DNA and RNA level profiles throughout the HPV genome for samples in Category 1. Each sample has two profiles, an RNA profile representing gene expression levels on an RPKM scale, and a DNA profile representing DNA content within the sample shown as copies per cell.

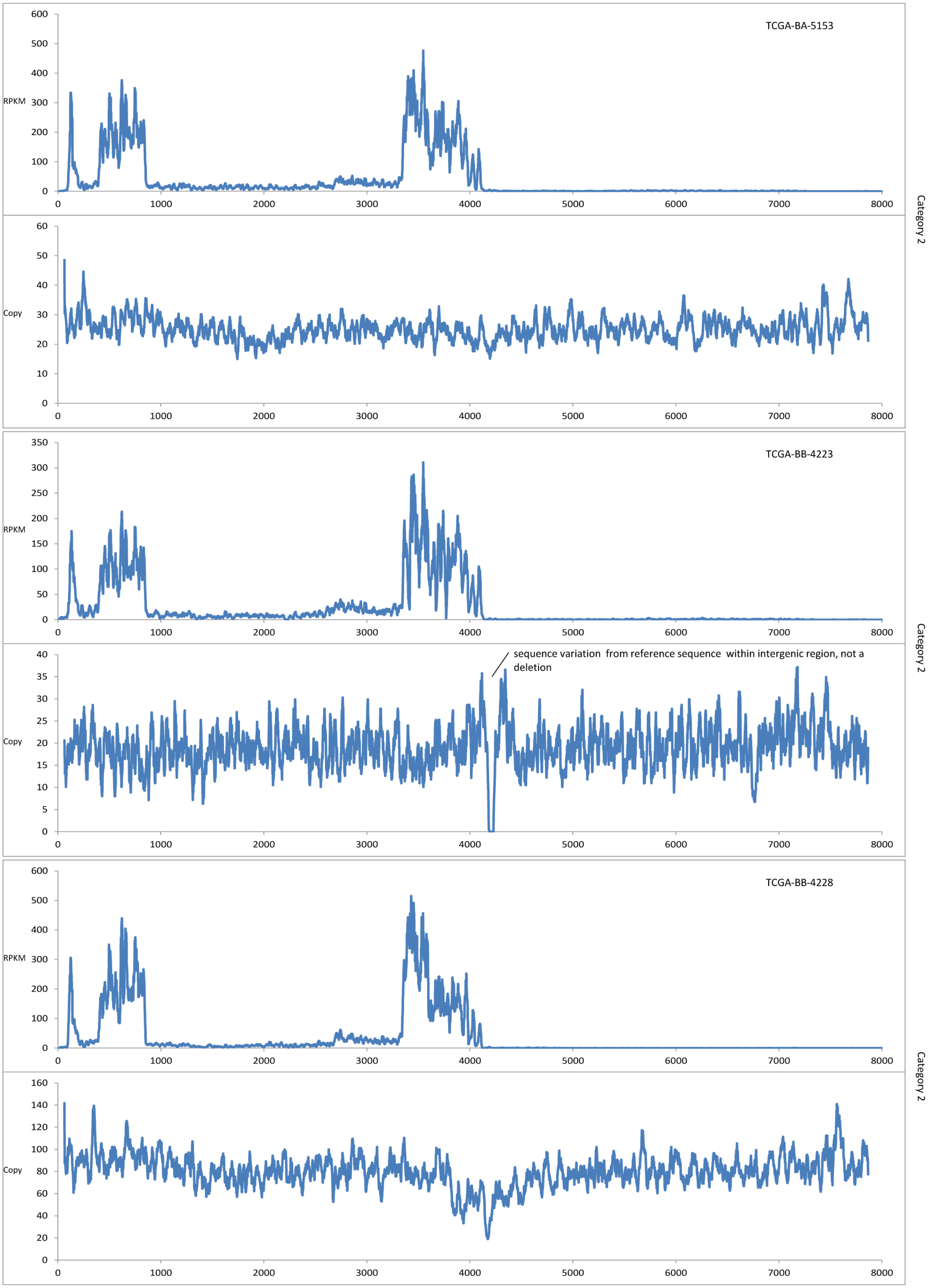
TCGA-BA-5153
RPKM
Copy
Category 2
TCGA-BB-4223
sequence variation from reference sequence within intergenic region, not a deletion
Category 2
TCGA-BB-4228
Category 2
0
1000
2000
3000
4000
5000
6000
7000
8000

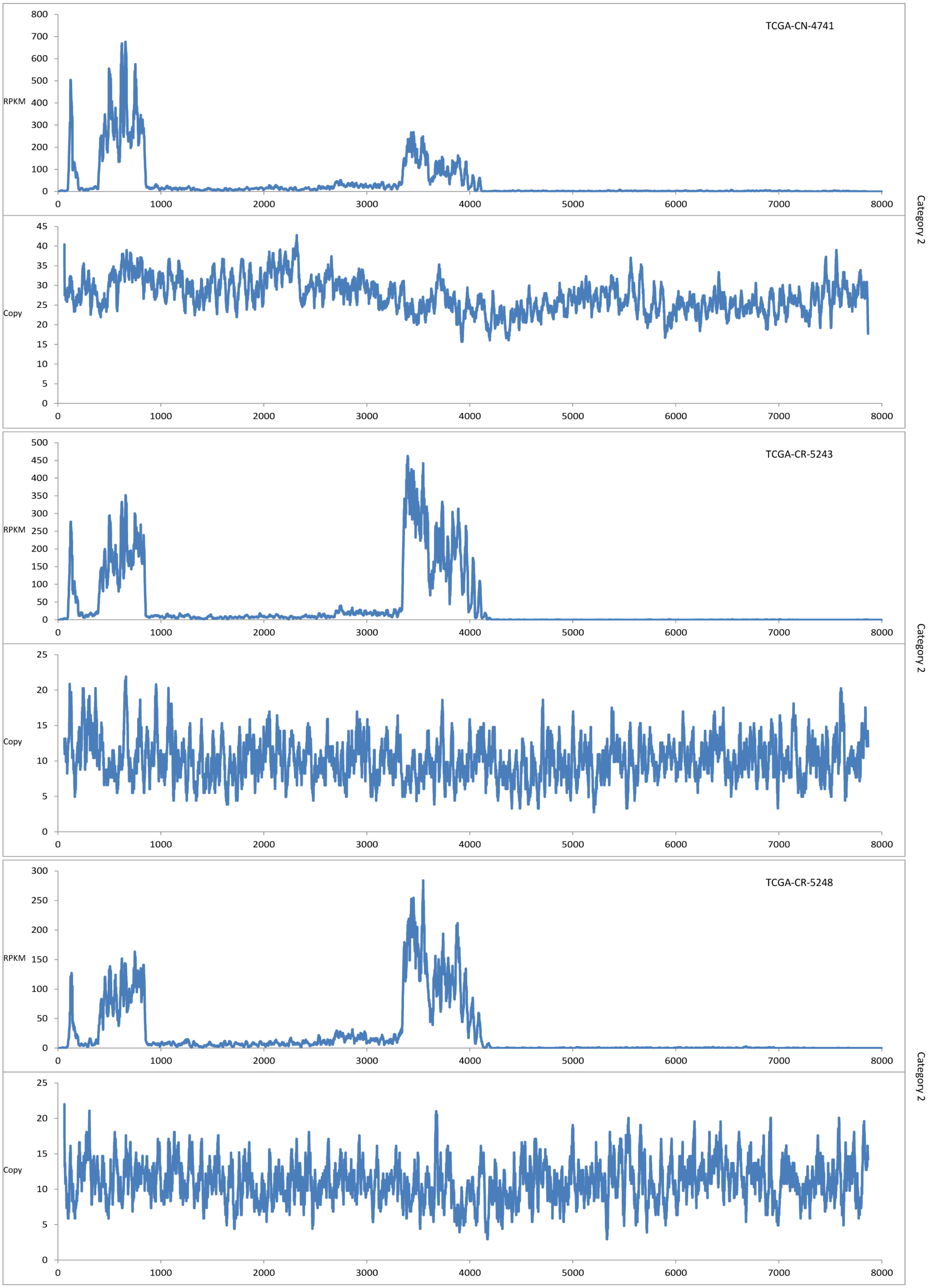
TCGA-CN-4741
RPKM
Copy
Category 2
TCGA-CR-5243
RPKM
Copy
Category 2
TCGA-CR-5248
RPKM
Copy
Category 2

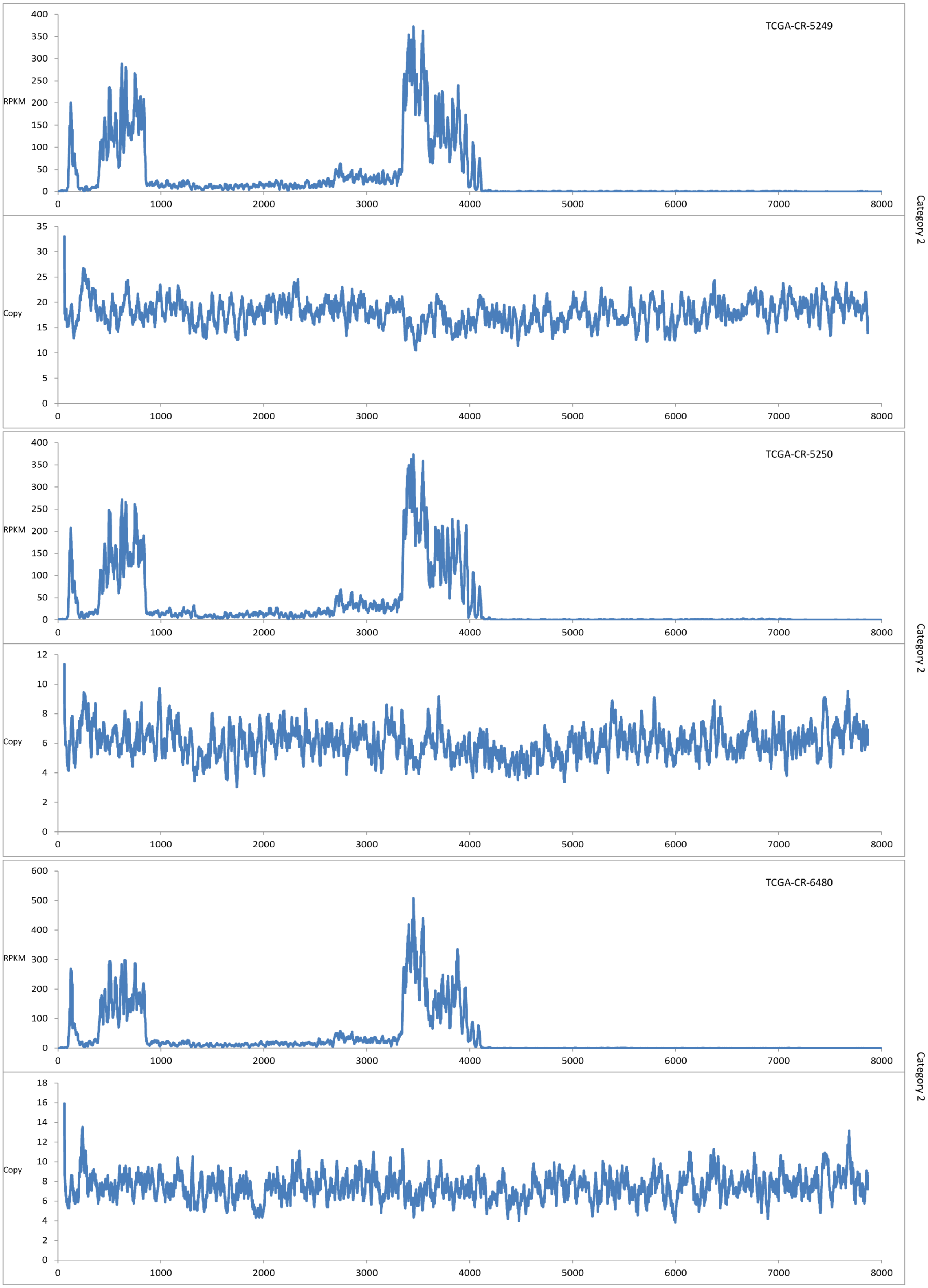
TCGA-CR-5249
RPKM
Copy
Category 2
TCGA-CR-5250
RPKM
Copy
Category 2
TCGA-CR-6480
RPKM
Copy
Category 2

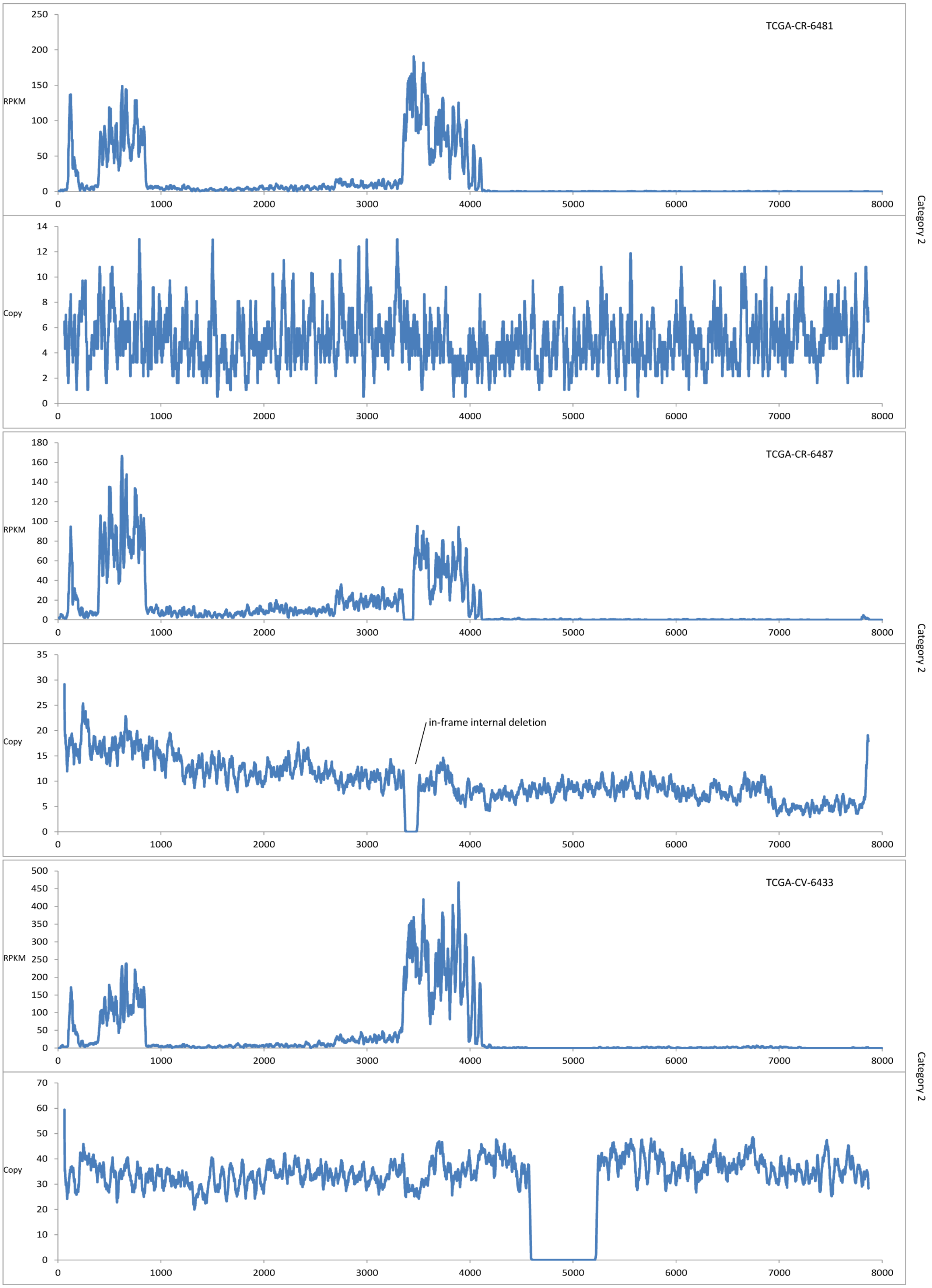

TCGA-CR-6481
RPKM
Copy
Category 2
TCGA-CR-6487
RPKM
Copy
in-frame internal deletion
Category 2
TCGA-CV-6433
RPKM
Copy
Category 2
0
1000
2000
3000
4000
5000
6000
7000
8000

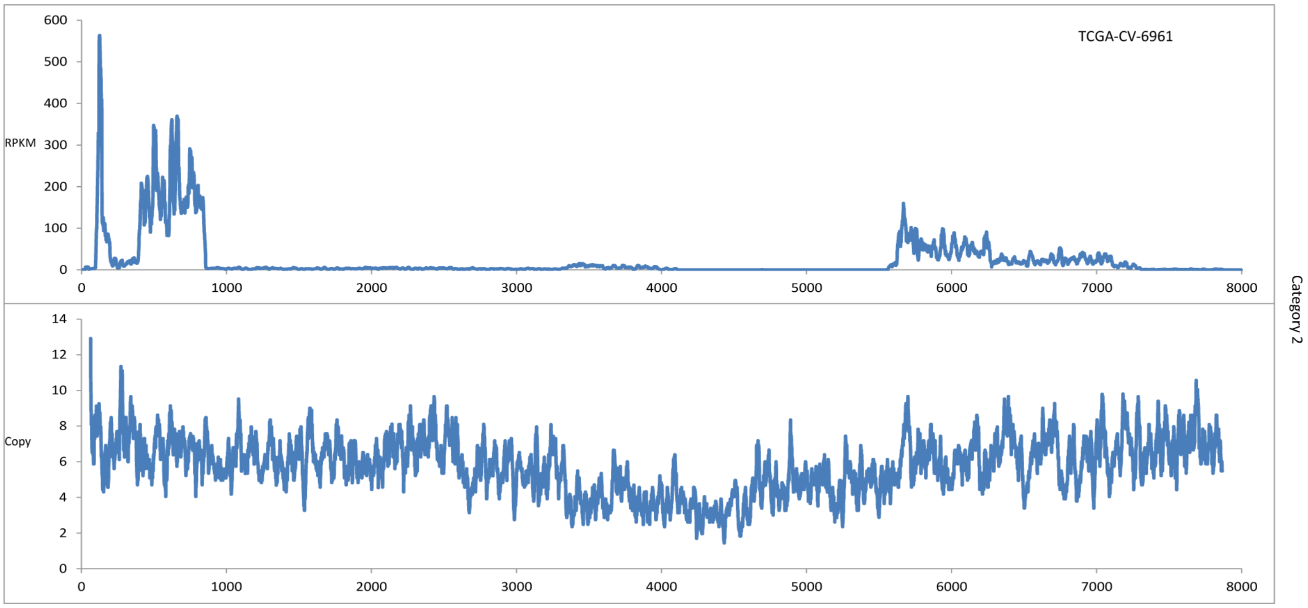

Figure S2: DNA and RNA level profiles throughout the HPV genome for samples in Category 2. Each sample has two profiles, an RNA profile representing gene expression levels on an RPKM scale, and a DNA profile representing DNA content within the sample shown as copies per cell.

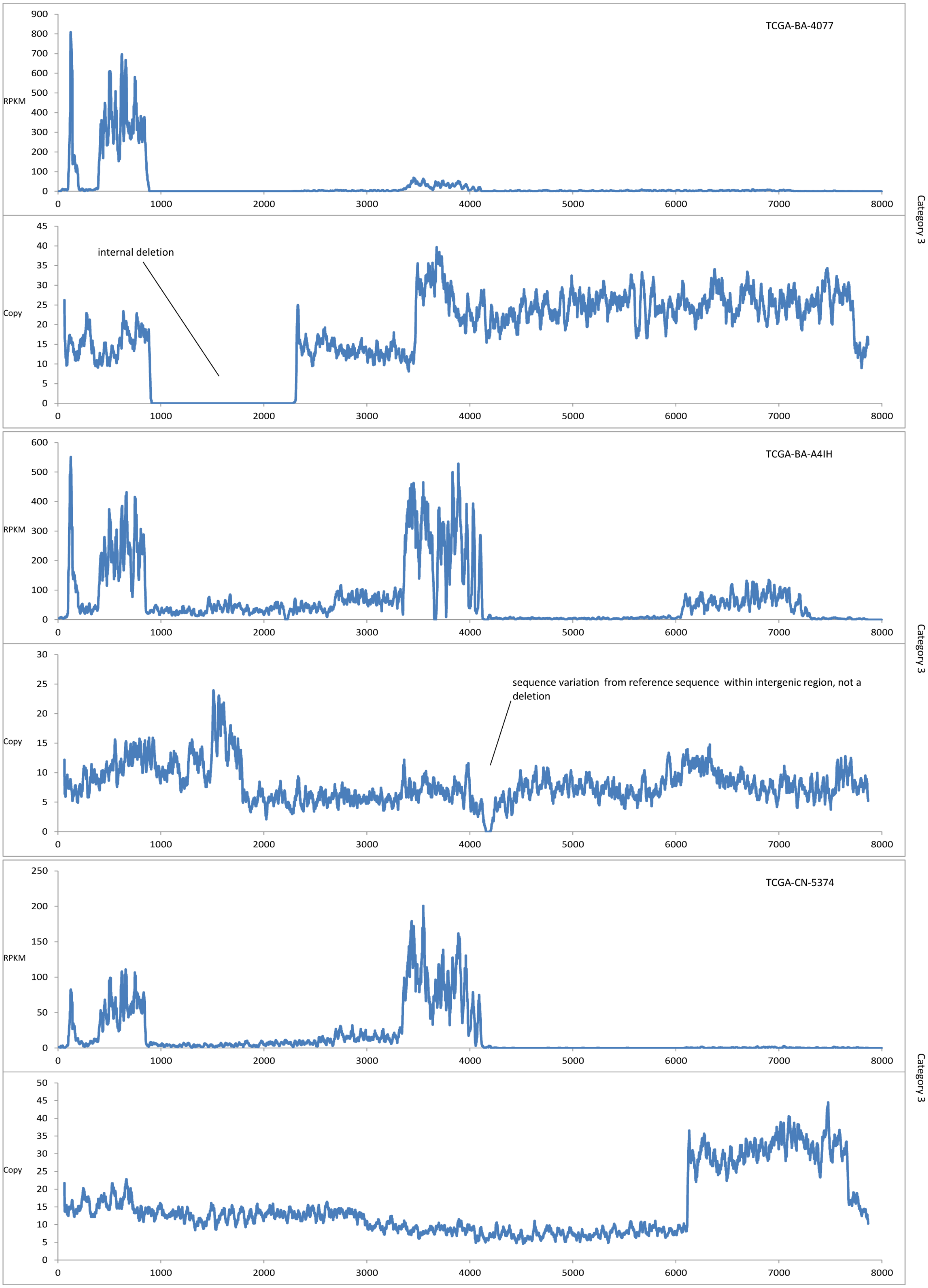
TCGA-BA-4077
RPKM
Copy
internal deletion
Category 3
TCGA-BA-A4IH
RPKM
Copy
sequence variation from reference sequence within intergenic region, not a deletion
Category 3
TCGA-CN-5374
RPKM
Copy
Category 3

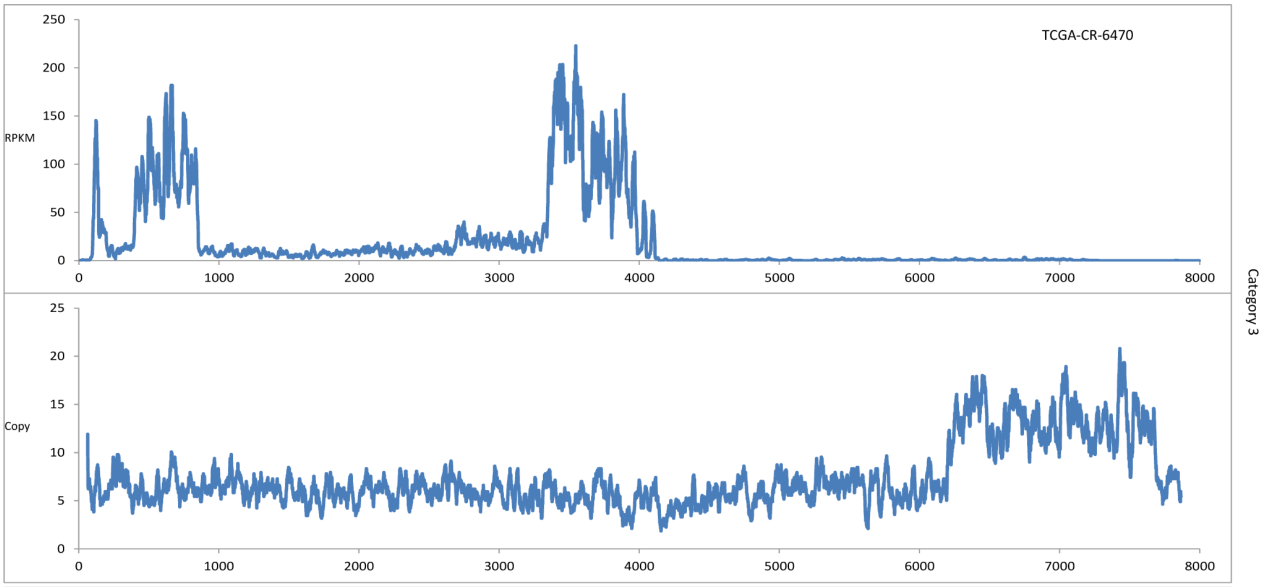
TCGA-CR-6470
RPKM
Copy
Category 3

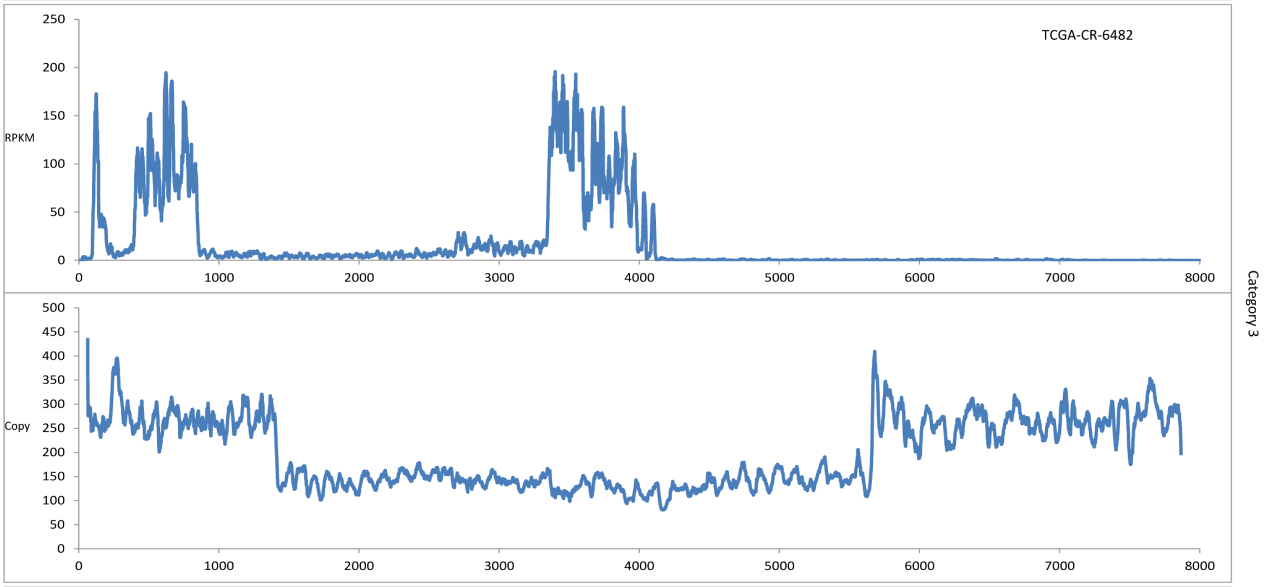
TCGA-CR-6482
RPKM
Copy
Category 3

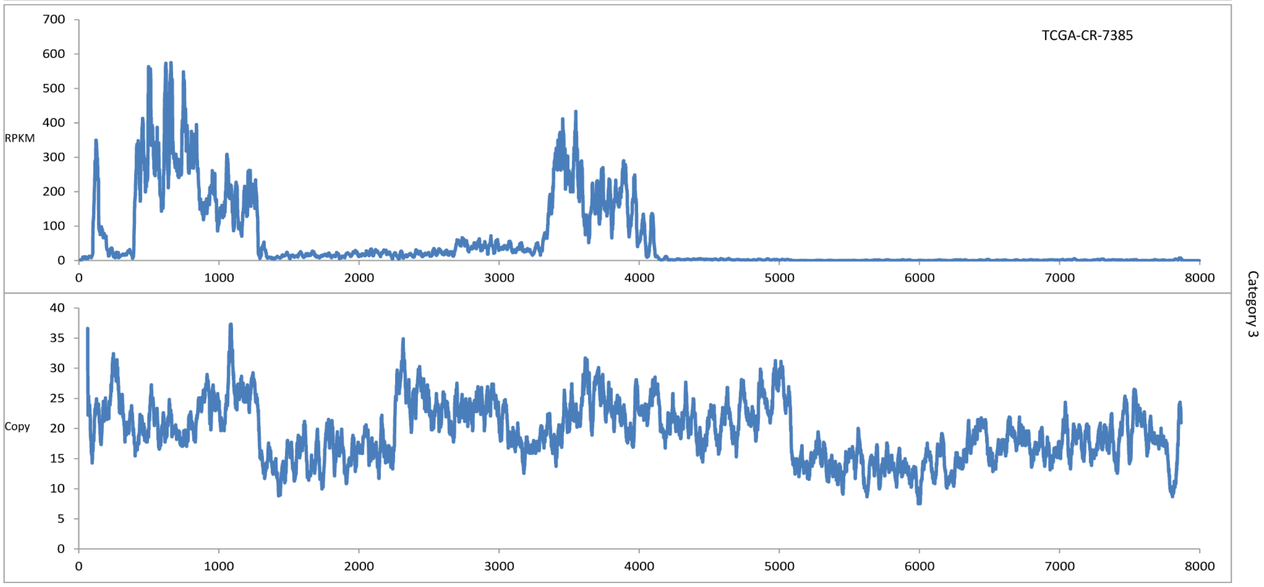
TCGA-CR-7385
RPKM
Copy
Category 3

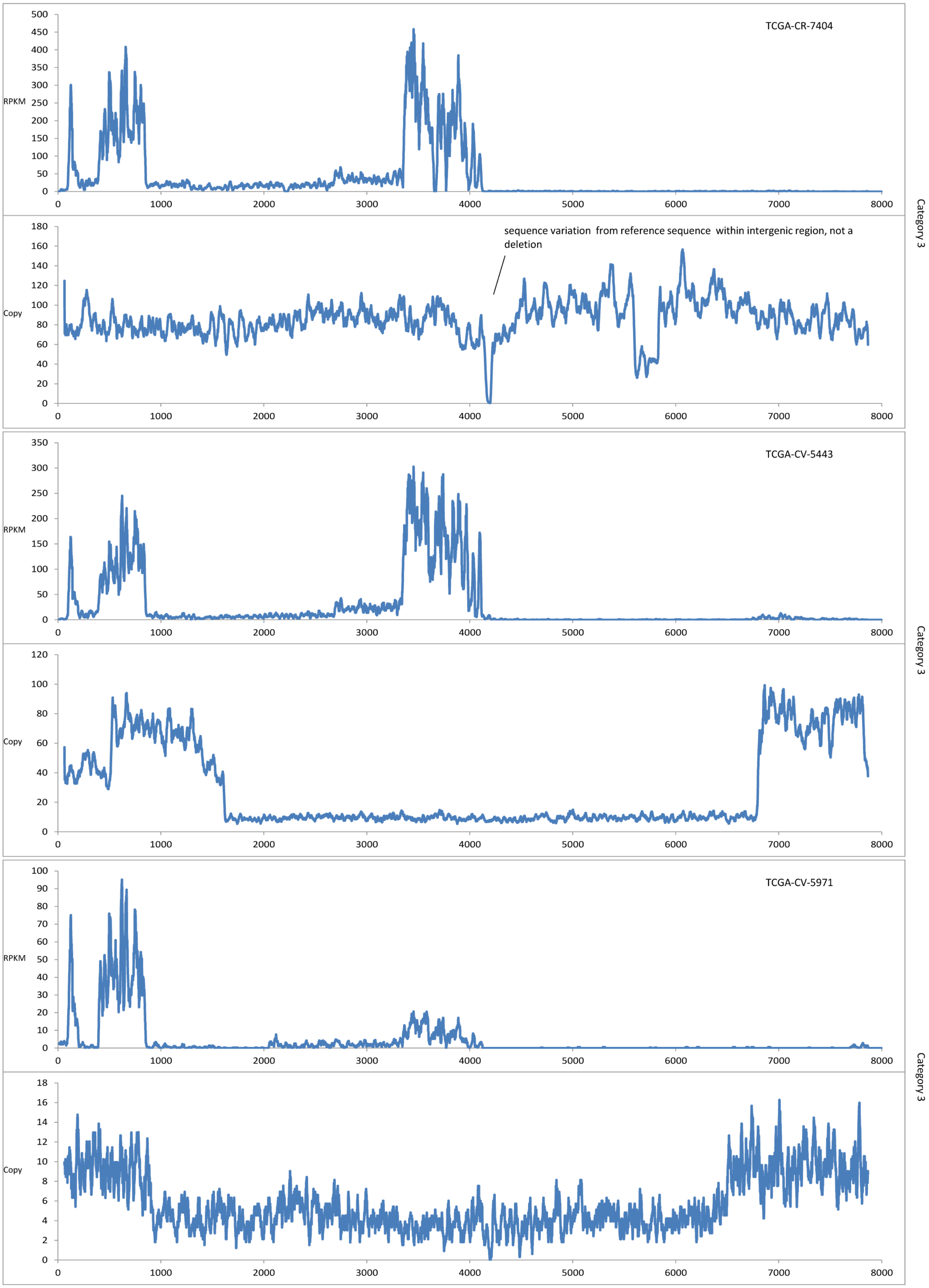
TCGA-CR-7404
RPKM
Copy
sequence variation from reference sequence within intergenic region, not a deletion
Category 3
TCGA-CV-5443
RPKM
Copy
Category 3
TCGA-CV-5971
RPKM
Copy
Category 3

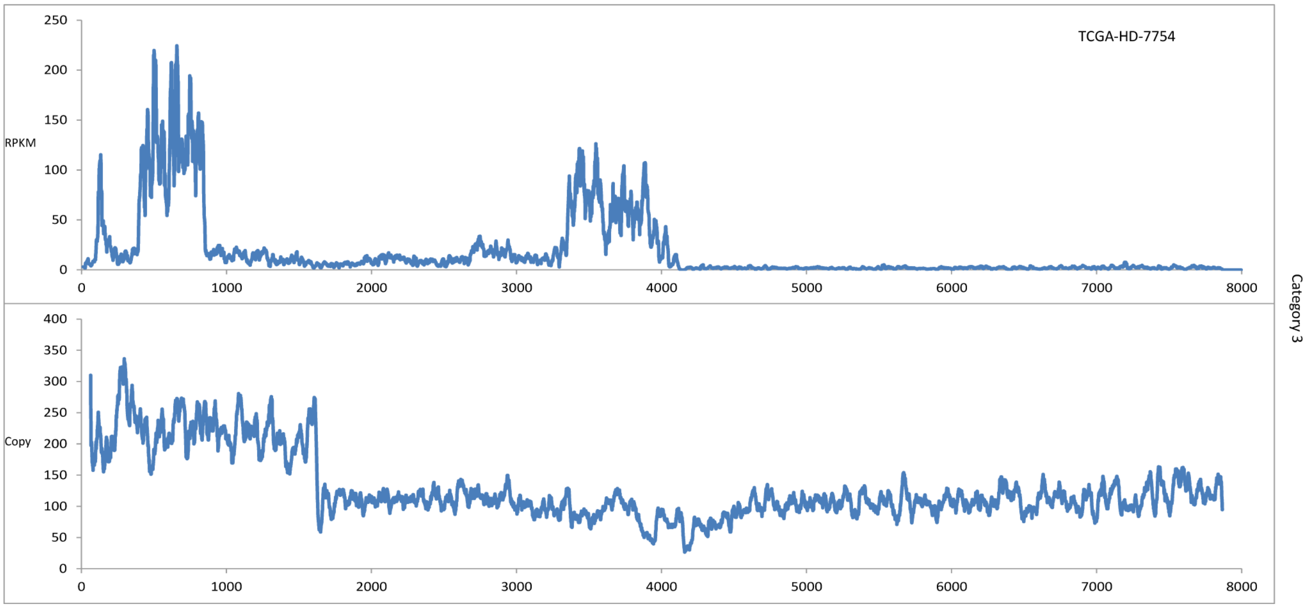

Figure S3: DNA and RNA level profiles throughout the HPV genome for samples in Category 3. Each sample has two profiles, an RNA profile representing gene expression levels on an RPKM scale, and a DNA profile representing DNA content within the sample shown as copies per cell.

Table 1- HPV Genomes for alignment. The viral names and IDs for genomes used in the initial alignment.

Table 2 - HNC HPV Types. The human papillomavirus types identified within head and neck cancer samples.

Table 3 - HNC HPV State Categories. A list of samples with the HPV16 categories for gene expression and genomic structure.

Table 4 - HPV-human jxn sites. A list of samples with HPV-human DNA junctions identified.

Table 5 - HNSC NR4A2,RAD51L1,TRPC4AP. TCGA gene expression data for three genes, NR4A2, RAD51L1, and TRPC4AP, which are each amplified in the first sample shown.

Table 6 - CR-6482 HPV-human jxn frgmts. A pairing of junction fragments with one end having HPV16 sequence and the other end having human sequence (shown in SAM format). Both junctions are listed.

Table 7 - CR-6482 HPV-human jxn1 rds. Hybrid reads for junction 1 with one end having HPV16 sequence and the other end having human sequence. The reads are position aligned with the consensus read sequence shown. Fragment identifiers and alignment are shown for each read.

Table 8 - CR-6482 HPV-human jxn2 rds. Hybrid reads for junction 2 with one end having HPV16 sequence and the other end having human sequence. The reads are position aligned with the consensus read sequence shown. Fragment identifiers and alignment are shown for each read.

Table 9 - CR-6482 hum-hum exc jxn frgm. A pairing of fragments covering an excision junction with each end having human sequence from distal locations (shown in SAM format).

Table 10 - CR-6482 hum-hum exc jxn rds. Hybrid reads for an excision junction with each end having human sequence from distal locations. The reads are position aligned with the consensus read sequence shown. Fragment identifiers and alignment are shown for each read.

Table 11 - 11. BA-4077 HPV-hum jxn frgmts. A pairing of junction fragments with one end having HPV16 sequence and the other end having human sequence (shown in SAM format). Both junctions are listed.

Table 12 - BA-4077 HPV-human jxn1 rds. Hybrid reads for junction 1 with one end having HPV16 sequence and the other end having human sequence. The reads are position aligned with the consensus read sequence shown. Fragment identifiers and alignment are shown for each read.

Table 13 - BA-4077 HPV-human jxn2 rds. Hybrid reads for junction 2 with one end having HPV16 sequence and the other end having human sequence. The reads are position aligned with the consensus read sequence shown. Fragment identifiers and alignment are shown for each read.

Table 14 - CV-5971 HPV-human jxn frgmt. A pairing of junction fragments with one end having HPV16 sequence and the other end having human sequence (shown in SAM format). Both junctions are listed.

Table 15 - CV-5971 HPV-human jxn reads. Hybrid reads for junction 1 and junction 2 are listed with one end having HPV16 sequence and the other end having human sequence. The reads are position aligned with the consensus read sequence shown. Fragment identifiers and alignment are shown for each read.
